# Modeling Thin Film Solar Cells: From Organic to Perovskite

**DOI:** 10.1002/advs.201901397

**Published:** 2019-11-07

**Authors:** Deli Li, Lin Song, Yonghua Chen, Wei Huang

**Affiliations:** ^1^ MIIT Key Laboratory of Flexible Electronics (KLoFE) Shaanxi Key Laboratory of Flexible Electronics (KLoFE) Xi'an Key Laboratory of Flexible Electronics (KLoFE) Xi'an Key Laboratory of Biomedical Materials & Engineering Xi'an Institute of Flexible Electronics Institute of Flexible Electronics (IFE) Northwestern Polytechnical University (NPU) Xi'an 710072 Shaanxi P. R. China; ^2^ Key Laboratory of Flexible Electronics (KLoFE) & Institution of Advanced Materials (IAM) Jiangsu National Synergetic Innovation Center for Advanced Materials (SICAM) Nanjing Tech University (NanjingTech) Nanjing 211816 Jiangsu P. R. China; ^3^ Key Laboratory for Organic Electronics & Information Displays (KLOEID), and Institute of Advanced Materials (IAM) Nanjing University of Posts and Telecommunications Nanjing 210023 Jiangsu P. R. China

**Keywords:** device models, organic semiconductors, perovskites, thin film solar cells

## Abstract

Device model simulation is one of the primary tools for modeling thin film solar cells from organic materials to organic–inorganic perovskite materials. By directly connecting the current density–voltage (*J*–*V*) curves to the underlying device physics, it is helpful in revealing the working mechanism of the heatedly discussed organic–inorganic hybrid perovskite solar cells. Some distinctive optoelectronic features need more phenomenological models and accurate simulations. Herein, the application of the device model method in the simulation of organic and organic–inorganic perovskite solar cells is reviewed. To this end, the ways of the device model are elucidated by discussing the metal–insulator–metal picture and the equations describing the physics. Next, the simulations on *J*–*V* curves of organic solar cells are given in the presence of the space charge, interface, charge injection, traps, or exciton. In the perovskite section, the effects of trap states, direct band recombination, surface recombination, and ion migration on the device performance are systematically discussed from the perspective of the device model simulation. Suggestions for designing perovskite devices with better performance are also given.

## Graphical abstract

Device model simulation is a macroscopic computer‐assisted tool for modeling organic and organic–inorganic hybrid perovskite solar cells. It simulates the underlying physical mechanisms of the electrical characteristics, such as space‐charge‐limited current, injection‐limited current, ohmic contact, short‐circuit current density, open‐circuit voltage, *J*–*V* hysteresis phenomena, power conversion efficiency in the present of surface recombination, trap/defect dependent recombination, or direct band recombination.

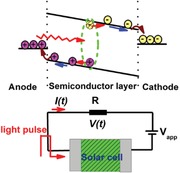

## 1 Introduction

The device model simulation is a macroscopic computer‐assisted technique that is increasingly being used to simulate the phenomenological characteristics of the thin film solar cells (i.e., the short‐circuit current density, the open‐circuit voltage with *V*_OC_ for short, and the fill factor).^1^, ^2^, ^3^, ^4^ These cells are typically fabricated by sandwiching a semiconductor layer of approximately tens or hundreds of nanometers between the two metal electrodes having different work functions.^5^, ^6^ The model is based on the metal–insulator–metal picture that originates from the early study of the conductivity in semiconductor and insulator problems.^7^ To understand the organic devices that are not suitable for Schottky models, the model was further developed.^8^, ^9^, ^10^ The model includes Poisson's equation, drift‐diffusion equations, and rate equations with corresponding boundary and initial conditions to model the underlying charge carrier processes. It distinguishes itself from the detailed balance theory^11^, ^12^ and the equivalent circuit theory^13^, ^14^ by a more informative and factual description of the performance of the cells. In general, the physical processes inside a solar cell can be divided into charge carrier generation, recombination, conduction, and collection. Each of these processes is closely related to the electrical characterization of the device. The advantage of the device model approach is that there is a direct link between the observable *J*–*V* curve and each of the associated microphysical processes. Moreover, Foster et al. analyzed the device model by using a combination of asymptotic and numerical techniques. This leads to an expression for *J*–*V* relationship as a function of the thermal voltage. Their results indicate that the device model simulation can accurately give the overall information of the thin film solar cells while maintaining quasi‐thermodynamic equilibrium.^15^

**Figure 1:**
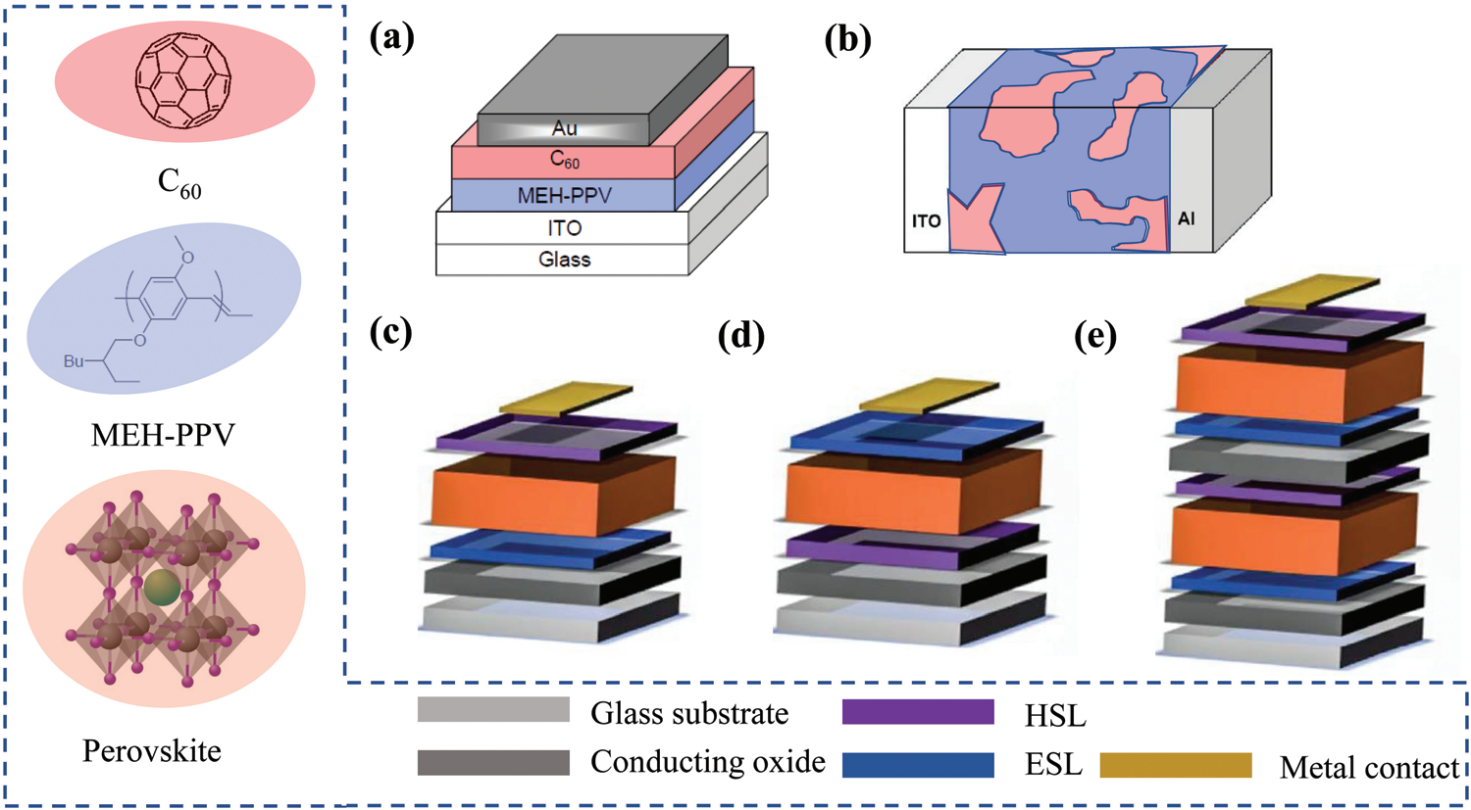
a) A bilayer heterojunction solar cell with the configuration of C_60_/MEH‐PPV/ITO. b) Bulk heterojunction solar cell with donor (cyan)/acceptor(red) dispersed between a transparent ITO electrode and an Al electrode. Reproduced with permission.^57^ Copyright 2004, Elsevier B.V. c) NIP PSCs with a conducting glass/electron selective layer (ESL)/perovskite/hole selective layer (HSL) configuration. d) The PIN with a conducting glass/HSL/perovskite/ESL configuration, which also referred to an inverted configuration. e) Tandem perovskite with a multijunction configuration, in which two or more bandgap‐matched absorbers are stacked. Reproduced with permission.^24^ Copyright 2017, American Association for the Advancement of Science.

This method has been successfully applied to the modeling of various organic solar cells (OSCs), such as organic single‐layer diodes,^16^, ^17^ bilayer OSCs,^2^ and organic heterojunctions.^18^, ^19^ Through a simulation of the corresponding microscopic electronic processes, the simulation reproduces many of the features of the *J*–*V* curve. Contact of the semiconductor with the electrode,^16^ space charge,^17^ exciton,^2^, ^18^ and electronic process at the junction^18^ have been shown to have a combined effect on the performance of the OSCs. Smith and co‐workers simulated a charge carrier injection in a plane‐parallel structure, such as a solar cell with structure of Al/MEH‐PPV [poly(2‐methoxy‐5‐(2‐ethylhexyloxy)‐1,4‐phenylvinylene)]/ITO.^9^ Thermal electron emission and tunneling of charge carriers, such as intraband tunneling and trap‐assisted tunneling at the contact of these devices, have been shown to be closely related to the charge in the semiconductor. Through successful fitting of the experimental results, the concept of the space‐charge‐limited current and the injection‐limited current is accurately understood.^20^ Blom and co‐workers incorporated bimolecular recombination, exciton processes, and a temperature‐ and field‐dependent generation mechanism of free charges into the device model.^1^ The effect of the physics of exciton dissociation and recombination on the performance of OSCs has been analyzed in detail. The device model plays an important role in elucidating the mechanism of exciton dissociation and recombination. Koster and co‐workers used the device model to simulate the charge carrier physical processes at the heterojunction; for example, with a C_60_/MEH‐PPV/ITO configuration (**Figure** **1**a), and bulk heterojunction solar cell with electron donor [poly(3‐hexylthiophene‐2,5‐diyl)] and electron acceptor [PCBM(1‐(3‐methoxycarbonyl)propyl‐1‐phenyl[6,6]C_61_))] dispersed between a transparent ITO electrode (Figure 1b).^19^ These early themes are common problems that all thin film devices would need to handle later.

Currently, tremendous efforts are put into developing ultrathin film perovskite solar cells (PSCs) based on semiconductors of organic–inorganic hybrid perovskites, such as MAPbX_3_ (X = Br, I, and Cl).^21^, ^22^, ^23^ Vapor‐deposited methods and solution‐processed methods have been applied to their preparation.^24^, ^25^ With the adoption of the perovskite materials and solution‐processed techniques, device structures of more complexity have been developed. They are planar architectures with an electron selective layer (ESL)/hole selective layer (HSL), particularly the NIP and PIN configurations in Figure 1c,d. Multijunction device architectures, as shown in Figure 1e, have also been hotly studied with layers piling one above another. Addictive and low dimensional microstructures in the perovskite layers are other ways to improve device performance, which further increases the complexity of the device. Through these material designs and device optimization, the power conversion efficiency (PCE) of the PSCs has quickly risen to 23.7% in less than ten years.^26^, ^27^, ^28^, ^29^ However, there is still much work to be done to achieve mass‐producible, priceless and pollution‐free energy from the PSCs. One of the crucial issues is the investigation for the working mechanisms, which is still in the starting states. A quantitative analysis theory of the device is still unavailable.

The device model theory in the OSCs can be used as a starting point for understanding the working mechanism and performance of PSCs. The device model can handle problems, such as solving the PCE limit of the cells, under the influence of the contacts to the electrodes, the properties of the semiconductor layers and the interfaces between layers.^30^, ^31^, ^32^ The PCE, *V*_OC_, and fill factor of the PSCs are sensitive to many of the properties of the ESL/HSL, such as doping density,^33^ valance band/conduction band,^34^ and charge mobility.^35^ They are relevant to the charge carrier collection and recombination in the perovskite layer. Charge transport, charge injection, collection, and recombination at the interface of PSCs are still under debate. The disparity of PCE to the Shockley–Queisser limit of ≈31% needs to be explained.^11^, ^12^ These problems are well handled by the device models. The device model method and its conclusions that were implemented in OSCs simulation have been gradually applied to the study of complex PSCs based on different functional layers. The layers usually have tightly correlated charge carrier processes, which makes the traditional processing method incompetent. For example, by employing the device model simulations, Wu and co‐workers investigated the bimolecular recombination and the trap‐assisted monomolecular recombination in meso‐structured perovskite solar cells under a steady state working condition. The most significant contribution for the *V*_OC_ and relatively high efficiency was attributed to the reduced bimolecular recombination.^36^ For poorly fabricated PSCs, considerable PCE loss was revealed to be related to the high trap density in bulk or at the ESL/perovskite interface.^37^

In addition to the traditional device model topics, many discoveries imply new physical processes that require much theoretical work and simulation. Ion migration is the most peculiar one in PSCs, which attracts much research interest.^38^ Both the experiments and first principle studies indicate that methylammonium ions (MA^+^) are easily excited with a reorientation energy barrier of ≈0.01 to 0.098 eV, which is low enough to be crossed at room temperature.^39^ The ions are believed intuitively to screen the external field and further influence the PCEs.^40^ In recent works, by coupling slow ion motions and the surface recombination at the ESL/perovskite, the device model was used by Frost et al. to explain the mysterious hysteresis phenomena that was observed in many perovskite cells.^41^ Reproduce the current density–voltage hysteric response and many of its features by using device model methods have an irreplaceable role in defining its cause. The morphology of perovskite thin film is another important issue in the preparation of PSCs. The grain boundary is the fine structure of the perovskite layers, and it plays an important role in determining the performance of PSCs.^42^ Defects introduced into grain boundaries often capture charge carriers, which lead to nonradiative recombination losses. Modification of the morphology of the thin film can assist the charge carrier collection, thus indicating microscopic charge carrier physical processes at the boundary, which is a topic of great research value.^43^, ^44^, ^45^ A detailed understanding of the grain boundaries and accurate modeling of the physical processes are essential to improve performance. The third new topic is the unique function of heterojunctions, which is a commonly used selective layer/perovskite structure shown in Figure 1c,d. The ESL,^46^ for example, is implemented to enhance charge collection by blocking holes. However, other charge carrier processes are introduced by the selective layer, which should be evaluated in a practical device design to avoid adverse effects on the charge collection.^47^

As far as we know, many review articles have made a proper analysis of the specific problems of PSCs, such as the manufacturing process, interface problems, material physical properties, and device structure.^42^, ^48^, ^49^, ^50^, ^51^, ^52^ However, a systematic macroscopic device model consideration of the thin film device is still lacking. This paper provides a systematic methodological overview of device model tools and their applications in thin film solar cell simulation, understanding, and design. We begin first by presenting the device model, followed by the implementation of the models into a typical organic thin film solar cell in Section 3. We include the simulation of organic devices because the influence of the space charge, interface, charge injection, trap, and excitons on the performance is the early topic of the device model simulation on the thin film cell. The results are helpful in simulating the organic–inorganic perovskite thin film solar cells. Following this, we show the application of the device model in typical organic–inorganic perovskite thin film solar cells in Section 4.1 in terms of the effects of the trap states, direct band recombination, surface recombination, and ion migration.

## 2 The Device Model

### 2.1 Metal–Insulator–Metal Picture

**Figure 2:**
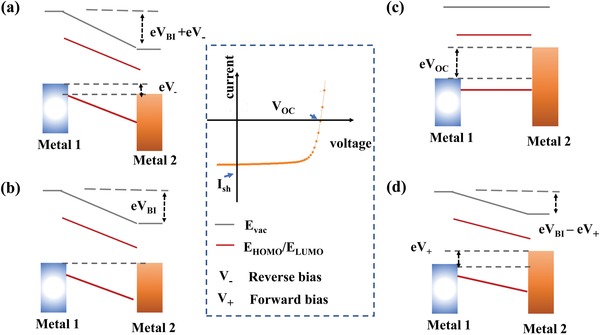
Metal–Insulator–Metal picture of the thin film device and schematic *J*–*V* curves of the solar cells with characteristics of short‐circuit current, *V*_OC_. The schematic the division of different work conditions, including a) reverse bias condition: the electric field is bigger than the build in voltage (*V*_BI_), b) short circuit condition: the photogenerated charges drift toward the contacts by the *V*_BI_, c) open circuit condition: the photogenerated charges recombine and the current becomes zero, and d) forward bias less than *V*_OC_: the internal field is weakened by the forward bias. Adapted with permission.^53^ Copyright 2004, Materials Research Society.

The thin film devices are described by using the metal–insulator–metal picture.^53^, ^54^, ^55^, ^56^ In the picture, the device is simplified as an electronic structure, and physical process of the carrier is determined by the parameters. The semiconductor is represented by the lowest unoccupied molecular orbital, which is short written by LUMO, and the highest occupied molecular orbital, which is short written by HOMO. LUMO and HOMO are the conduction band and valence band, respectively, as shown in **Figure** **2**. The bandgap of the semiconductor is defined by the energy difference of LUMO and HOMO. The electrodes are represented by the Fermi levels. The picture provides a rectifying behavior of the intrinsic semiconductor device. Under the condition of thermal equilibrium, due to the asymmetry of the work function between the cathode and anode, uniform band bending is produced, and a built‐in electric field is formed. Under working conditions, an external voltage is applied to the equipment, and physical processes, such as charge generation, transportation and collection, occur under a built‐in electric field and external electric fields. It should be pointed out that defects and traps are introduced by local states in some problems to consider their effects on carrier conduction and recombination.

Upon illumination, the *J*–*V* curves reflect the electrical response of the device to the applied voltage, which constitutes the main problem to be simulated by the device model. Figure 2a shows the application of a reverse bias voltage. The collection of charge carriers is enhanced under a reverse bias voltage, i.e., the applied voltage is applied to the built‐in voltage. Because the injected dark current is very small, the total current is composed mainly of the collecting current. Figure 2b shows the short circuit without applying voltage, and only the built‐in electric field that formed by the difference in the work function of the electrodes is distributed throughout the device. Under illumination, the generated charge carriers drift to the contacts in the built‐in electric field, which resulted in a short‐circuit current. Under the open circuit situation (Figure 2c), a voltage is established to offset the built‐in electric field without the current flowing through the device. As a result, the charge carriers relax to the ground state by recombination radiatively or nonradiatively. In addition, close to the *V*_OC_, charges can be effectively injected from contacts into semiconductors, thus leading to dark currents that should not be ignored. Therefore, the recombination velocity and the dark current density have a significant influence on the shape of the *J*–*V* curves at the voltage near the *V*_OC_. Figure 2d shows the situation with a forward bias that weakens the internal electric field. When the solar cell works under a positive bias and the applied voltage is less than *V*_OC_, the charge collection of the internal electric field will still occur. However, if the forward bias is greater than *V*_OC_, then the charge carriers recombine, and the photovoltaic effect will not occur.

The *J*–*V* curve is directly related to the electronic process, which is determined by the specific structure and material of solar cells. Under standard conditions, *V*_OC_, the short‐circuit current and the fill factor (i.e., the ratio of actual maximum power output to the theoretical power output defined by *V*_OC_ and the short‐circuit current products) are common characteristics of photovoltaic cell performance. Therefore, by simulating the electronic process, the device model can predict the characteristics of solar cells. On the other hand, by fitting the characteristics of solar cells, the device models can be used to reveal the electronic processes that have not been studied.

The maximum *V*_OC_ predicted by the metal–insulator–metal picture is the difference in the work function between the two electrodes. However, due to the existence of a multilayer structure, there is a high degree of complexity in understanding the origin of the *V*_OC_ in thin film solar cells. In practical applications, the *V*_OC_ has deviated from the metal–insulator–metal picture in a wide variety of device designs. The contacts of the semiconductor to the electrodes should be considered. Ideally, the lineup of the Fermi energy to the conduction band/valance band allows a *V*_OC_ with a value of the bandgap *E*_g_, which is defined by the threshold for the absorption of light. In thermodynamic equilibrium, the radiation process of solar cells must be in equilibrium with the light absorption process as the inverse process. As a result, *V*_OC_ loss is inevitable.^58^ The detailed balance between luminescent emission and absorption gives the well‐known Shockley–Queisser limit.^11^, ^12^ In addition, the contact, defects, carrier mobility, and surface recombination also have destructive effects on photovoltaic effects, which should be described fully from perspective of the device model.^59^

Based on the consideration of OSCs and PSCs, empirical conclusions have been drawn to illustrate the effect of the contacts on *V*_OC_. In the nonohmic condition (i.e., a charge carrier injection energy barrier larger than 0.3 eV), no charge carrier is injected into the energy disorders states, and the trap states or mid‐gap states are unoccupied. *V*_OC_ has a linear relationship with the work function of metals. Under the ohmic condition (i.e., a charge carrier injection energy barrier smaller than 0.3 eV), due to the charge localization at the interface, the work function of the contact is aligned with the quasi‐Fermi level. The local charge density leads to a dipole and *V*_OC_ loss. *V*_OC_ is affected by the energy level of the disordered state, trapped state or middle gap state. The work function of metal has little effect on the *V*_OC_ value.

Scharber et al. studied the *V*_OC_ 60 of various organic heterojunctions under ohmic contact conditions. They found an empirical relationship, such as

$$eV_{\mathrm{oc}}=E_{g}-0.3eV$$

where *E*_g_ is the donor–acceptor energy gap, and *e* is the elementary charge. The empirical formula shows that the *V*_OC_ is mainly determined by the donor–acceptor gap of semiconductors with a deviation of 0.3 V. Blakesley and Neher explained that the loss of 0.3 V *V*_OC_ was caused by the energy disorder in the organic layer.^61^ This was consistent with the reported energy levels of HOMO and LUMO, which broadened with the energy range of 0.1–0.2 eV.^62^, ^63^ Therefore, it can be illustrated that quasi‐Fermi levels of these devices are determined mainly by the donor–acceptor energy gap of semiconductors and energy disorder in the case of an open‐circuit voltage condition.

In PSCs, it is found that the contact between the perovskite layer and HSL and ESL is closely related to *V*_OC_. The parameters of the devices are listed in **Tables** **1** and **2**. For devices with a structure of the MAPbI_3_ halide perovskite, the perovskite layer is connected with a diverse range of ESL and HSL. In Table 1, the *V*_OC_ and the bandgap width are compared. A universal voltage loss Δ*V*, which is defined as *E*_g_/*e* − *V*_OC_, is found to be ≈0.45 V, according to the 1.5 eV energy gap of MAPbI_3_ halide perovskite. The electron/hole injection barriers Δ*E*_1_ (the energy differences of conduction band‐edge of ESL with LUMU of the perovskites) and Δ*E*_2_ (the energy difference HOMO of HSL with the HOMO of the perovskites) are less than 0.3 eV, which results in ohmic contacts. Similarly, an empirical relationship is drawn as

$$eV_{\mathrm{oc}}=E_{g}-0.45eV$$

**Table 1:** The *V*_OC_ loss Δ*V* = *E*_g_/*e* − *V*_OC_ for devices based on MAPbI_3_, the LUMO/HOMO of MAPbI_3_ are (−3.9/−5.4) eV. *E*_C_ is the conduction band of ESL, and *E*_V_ is the valence band of HSL. Δ*E*_1_ and Δ*E*_2_ are the injection barriers for electron and hole respectively

*V*_OC_ [V]	ESL	*E*_C_ [eV]	HSL	*E*_V_ [eV]	Δ*E*_1_/Δ*E*_2_ [eV]	Δ*V* [V]	Ref.
1.04	TiO_2_	−4.0	P‐TAA	−5.14	0.1/0.26	0.46	[qv: 64a,c]
0.92	TiO_2_	−4.0	PF8‐TAA	−5.44	0.1/−0.4	0.58	[qv: 64c]
1.04	TiO_2_	−4.0	PIF8‐TAA	−5.51	0.1/−0.11	0.46	[qv: 64c]
1.09	TiO_2_	−4.0	P3HT	−5.0	0.1/0.4	0.41	[qv: 64d]
1.01	TiO_2_	−4.0	Spiro‐OMETAD	−5.22	0.1/0.08	0.49	[qv: 64b]

**Table 2:** The *V*_OC_ loss Δ*V* = (*E*_C_ − *E*_V_)/*e* − *V*_OC_ for devices based on MAPbBr_3_, the LUMO/HOMO of MAPbBr_3_ are (−3.4/−5.6) eV. *E*_C_ is the conduction band of ESL, and *E*_V_ is the valence band of HSL. Δ*E*_1_ and Δ*E*_2_ are the injection barriers for electron and hole respectively

*V*_OC_ [V]	ESL	*E*_C_ [eV]	HSL	*E*_V_ [eV]	Δ*E*_1_/Δ*E*_2_ [eV]	Δ*V* [V]	Ref.
1.29	TiO_2_	−4.0	P‐TAA	−5.14	0.6/0.46	−0.15	[qv: 64a,c]
1.36	TiO_2_	−4.0	PF8‐TAA	−5.44	0.6/0.16	0.08	[qv: 64a,c]
1.40	TiO_2_	−4.0	PIF8‐TAA	−5.51	0.6/0.09	0.11	[qv: 64a,c]
1.20	TiO_2_	−4.0	Spiro‐OMETAD	−5.22	0.6/0.38	0.02	[qv: 64a,b]
1.61	ICBA	−3.7	PEDOT‐PSS	−5.3	0.3/0.3	0.01	[qv: 64e]
1.38	PCBM	−3.9	PEDOT‐PSS	−5.3	0.4/0.3	0.02	[qv: 64e]

The 0.4–0.6 V *V*_OC_ loss can be explained by the mid‐gap state of 0.58 eV energy deeper away from the edge of the conduction band. Mid‐gap states may be induced by the iodide ion, MA^+^, or defects at the grain boundary. The other destructive factors mentioned above should be checked under the simulation of the device model. It is proposed that the limitation of the defect state and the defect state combination must be overcome to increase the open circuit voltage of devices using MAPbI_3_ halide perovskites.

For devices with MAPbBr_3_ halide perovskite, the perovskite layer is connected with a diverse range of ESL and HSL. As shown in Table 2, it is not difficult to find that *V*_OC_ and the contact are closely related but are not related to the bandgap of the MAPbBr_3_ halide perovskite. Voltage loss Δ*V* is defined as (*E*_C_ − *E*_V_)/*e* − *V*_OC_ for a clearer indication of the correlation. A universal voltage loss Δ*V* less than 0.15 V is found according to the difference in the conduction band (*E*_C_) of ESL to the valence band (*E*_V_) of HSL. Both of the charge carrier injection barriers Δ*E*_1_ (the energy differences of the conduction band‐edge of ESL with LUMU of the perovskites) and Δ*E*_2_ (the energy difference HOMO of HSL with the HOMO of the perovskites) are ≈0.3 eV, which results in nonohmic contacts. In addition, the *V*_OC_ is determined by the conduction band of ESL and the valence band of HSL

### 2.2 Basic Equations

#### 2.2.1 1D Continuity Equations and Poisson's Equation

The continuity equation and Poisson's equation are the basis of establishing the physical devices model. The continuity equation (Equation (1)) gives the physical law constraints to ensure the conservation of particles, and Poisson's equation (Equation (2)) provides the correct electrical response of the device to the current carrier motion

(1) $$\frac{\partial }{\partial t}N_{n\left(p\right)}\left(x,t\right)\mp \frac{1}{q}\frac{\partial }{\partial x}J_{n\left(p\right)}\left(x,t\right)=G\left(x,t\right)-R\left(x,t\right)$$

(2) $$\frac{\partial^{2}\psi (x,t)}{\partial x^{2}}=-\frac{q}{\varepsilon_{0}\varepsilon_{r}}\left[N_{p}(x,t)-N_{n}(x,t)\right]$$

where *N*_p_(*x*,*t*), *N*_n_(*x*,*t*) are the free hole and electron density, *J*_p_(*x*,*t*), *J*_n_(*x*,*t*) are hole, electron current density, ψ(*x*,*t*) is the electrostatic potential, *G*(*x*,*t*) and *R*(*x*,*t*) are the particle generation rate and recombination rate, respectively. They are coordinate *x* dependent. ε_0_ and ε_r_ are the dielectric constants.

When the steady state is reached, the physical quantity does not change with time, and the continuity equation of charge carriers becomes

(3) $$\mp \frac{1}{q}\frac{\partial }{\partial x}J_{n(p)}(x,t)=G(x,t)-R(x,t)$$

*J*_p_(*x*,*t*), *J*_n_(*x*,*t*) are given by drift‐diffusion equations in Section 3.2.2. *G*(*x*,*t*), *R*(*x*,*t*) and the boundary conditions decided by the physical processes in the specified thin‐film cells that are under simulation. For example, the recombination items *R*(*x*) may be a single molecular recombination, a bimolecular recombination or a trap‐assisted surface recombination depending on the physical properties of the simulated cells. These items will be considered in the application sections of the device models, which are given in Sections 3 and 4.1 in this review.

Equations (1) and (3) have been used in modeling the steady state condition, depending on the simulation methods that were used to solve the equations.^19^, ^65^, ^66^ In transient problems, Equation (1) gives consistent results.^2^, ^67^, ^68^

#### 2.2.2 Drift‐Diffusion Equations of the Current Density

The drift‐diffusion form for current density is usually written as

(4) $$J_{n(p)}(x,t)=\mu_{n(p)}\left(qEN_{n(p)}\pm kT\frac{\partial }{\partial x}N_{n\left(p\right)}\right)$$

where *µ*_n(p)_ is the electron/hole mobility, *k* is Boltzmann's constant, *T* is the temperature, and the diffusion constant is substituted by the Einstein relation *D*_n(p)_ = *µ*_n(p)_ *kT*/*q*. The relation gives the mobility of the electrons/holes in terms of its diffusion coefficient and the environment temperature.

In the presence of traps, the drift‐diffusion equation is considered to be invalid, and the charge carrier transfer between the trap state and the conduction band will hinder the charge carrier movement. Approaches have been developed to deal with these problems. Sokel et al. divided the charge carrier into free charge transport carriers and trap charge carriers.^10^ The free charge transport carriers are described by the drift‐diffusion equation, and trap charge carrier dynamics are treated by the rate equation. The rate equation systems for trapped holes and trapped electrons are

(5) $$\frac{\partial n_{t}^{+(-)}}{\partial t}=T_{p(n)}-R_{n(p)}$$

(6) $$T_{p(n)}=t_{p(n)}N_{p(n)}\left(N_{t}^{+(-)}-n_{t}^{+(-)}\right)$$

(7) $$R_{p(n)}=r_{p(n)}N_{p(n)}n_{t}^{-(+)}$$

where $n_{t}^{+(-)}$ is the trapped hole (electron) density, *t*_p(n)_ is the trapping constant for holes (electrons), and $N_{t}^{+(-)}$ is the density of available hole (electron) traps, which are neutral when empty. The hole trapping *T*_p(n)_ is then proportional to the density of holes (electrons) and the density of unoccupied hole (electron) traps. By adding the trapping and detrapping dynamics, the equation system is enlarged twice.

In some cases, enlarging the dynamic equation set is not an ideal method of dealing with trap problems. This method requires more computer resources and more information, such as capture constants, which should be provided before simulation. One alternative method is to handle the charge transport with traps approximately. The mobility‐edge density of the state model is used to derive the approximate charge transport with traps.^8^, ^69^ In the approximation, thermal equilibrium is established between the conducting state and the trap state; by doing so, the drift‐diffusion equations with the corrected free charge density (hole for example) are written by

(8) $$J_{p}(x)=\frac{q}{\beta }{\left(\frac{N_{p}(x)}{N_{t}}\right)}^{\beta -1}\left[\mu_{p0}N_{p}(x)E-\beta D_{p}\frac{\partial N_{p}(x)}{\partial x}\right]$$

where β = (*kT* + *E*_t_)/*kT*), *E*_t_ is the characteristic trap energy relative to the *E*_HOMO_.

#### 2.2.3 Boundary Conditions for Continuity Equations

It is necessary to specify boundary conditions on the contact and interface between adjacent layers and to achieve the self‐consistent solution of the system by using the continuous equation and Poisson's equation, which formed from Equations (1) to (8). Charge carrier density boundary conditions and surface current boundary conditions are used in the device model.

*Charge Carrier Density Boundary*: For the steady state problems, thermodynamic equilibrium is assumed across the device. According to Boltzmann statistics, charge carrier densities are given as follows

(9) $$n(x=0)=N_{c}\exp\left(\frac{E_{\mathrm{F1}}-E_{c}}{k_{B}T}\right)$$

(10) $$p(x=b)=N_{v}\exp\left(\frac{E_{V}-E_{\mathrm{F2}}}{k_{B}T}\right)$$

(11) $$n_{i}(T)=\sqrt{N_{c}N_{v}}\exp\left(-\frac{E_{g}}{2k_{B}T}\right)$$

where *k*_B_ is the Boltzmann constant, *T* is the absolute temperature, *E*_F0_ and *E*_F1_ are the fermi‐energy of the contact at the interface denoted by *x* = 0 and *x* = b, *N*_c_ and *N*_v_ are the conduction band density of states valence band density of states, respectively, and *n*_i_ is the intrinsic carrier density.

At the ohmic contact, the states of the conduction band are fully occupied, and the charge carrier density boundary reduced to

(12) $$\begin{array}{l} n(x=0)=N_{c}\\ p(x=0)=N_{v}\exp\left(\frac{-E_{g}}{k_{B}T}\right)\\ n(x=b)=N_{v}\\ p(x=b)=N_{c}\exp\left(\frac{-E_{g}}{k_{B}T}\right) \end{array}$$

*Current Density Boundary*: The boundaries can also be alternatively modeled by specifying the particle currents at the contacts. The particle current components are determined by the current flow mechanism at the interface. In solar cell devices, there are two particle current components, a thermionic injection and a collection current that are the time‐reversed processes of thermionic injection. Specifically, consider the electron current at the metal/organic interface

(13) $$j_{n}=-j_{c}+j_{\mathrm{th}}$$

the thermionic recombination current is

(14) $$j_{\mathrm{th}}=A*T^{2}e^{-E_{b}/kT}$$

With A* as the effective Richardson's constant, and *E*_b_ = *E*_c_ − *E*_f_. The interface collection current directly proportional to the electron density at the interface is as follows

(15) $$j_{c}=qk_{c}n$$

The kinetic coefficient *k*_c_ is determined by the detailed balance between thermionic injection and interface collections without illuminations. It has the form of

(16) $$k_{c}=\frac{A*T^{2}}{qn_{0}}$$

where *n*_0_ is the density of states. Equation (16) presents the relationship between the kinetic coefficient *k*_c_, which is also commonly named the surface recombination velocity, and the effective Richardson's constant A*. For example, with a charge carrier state density of 10^21^ cm^−3^ and Richardson's constant, the kinetic coefficient reads

(17) $$k_{c}=\frac{120A{\mathrm{/cm}}^{2}/k^{2}\cdot {\left(300k\right)}^{2}}{1.6\times 10^{-19}C\cdot 10^{21}{\mathrm{/cm}}^{3}}=6.75\times 10^{4}\mathrm{cm}\;s^{-1}$$

*Comments*: Under normal work conditions, the light‐induced approximate charge carrier density Δ*n* of 10^13^ cm^3^ to 10^15^ cm^3^. The light‐injected current is just a disturbance to the carrier density at the boundary at the ohmic contact, for which Δ*n*/*n*_0_ is a minor number compared to $e^{-E_{b}/kT}>e^{-0.3eV/0.026eV}\approx 10^{-5}$. In most cases, the carrier density boundary with thermal equilibrium described by Equations (9) to (10) are used to model the contacts at the boundary conditions to simulate solar cells.

*Boundary Conditions on the Interface between Adjacent Layers*: The boundary conditions mentioned above are simplified when the actual cells are modeled. For real solar cells, there are interfaces between the multilayer structures. Some of the most important interfaces are the donor/acceptor interface of OSCs, the interface of heterojunction in OSCs and the interface between ESL/HSL and the perovskite layer in PSC. Specific boundary conditions must be established at the interfaces to connect different layers by modeling the physical processes at these boundaries. The detailed balance theory of the thermionic injection current and the recombination current provides a basis for dealing with this problem. Generally, the thermionic injection current and the recombination current are used to simulate the movement of charges across the interface. However, in some cases, the processes of the charge at the interface are very complicated by quantum tunneling, a defective state effect, and excitons playing a vital role in determining the electrical characteristics of devices. The tunneling injection current, recombination current in the presence of traps, and the exciton of physical process have been studied intensively in OSCs; some of the results will be discussed in Section 3.3.

For PSC, the precise boundary conditions of the interface between ESL/HSL and the perovskite layer are needed to ensure the accurate modeling of the current collection. The boundary conditions are similar to the charge carrier density boundary or the current density boundary mentioned above. In some models, the charge injection barrier electron beams are redefined by conduction band/valence band differences between ESL/HSL and perovskite layers, and the effect of bands on the device performance is studied. Whether quantum tunneling can be neglected is still a question, and the effect of the interface defects will be discussed in Section 4.4.

#### 2.2.4 Boundary Conditions for Poisson's Equation

**Figure 3:**
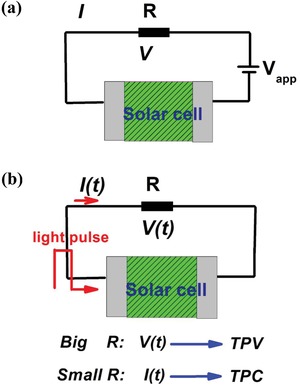
Schematic representation on the circuit. a) Steady state. b) The transient photocurrent (TPC)/photovoltage (TPV).

*Steady State*: In most cases, thin film cells are characterized by the *J*–*V* curve. During *J*–*V* measurement, a set of current densities are recorded with a set of applied scanning voltages at a constant scanning rate. The slow scanning rate ensures that cells operation at a steady state. In modeling the *J*–*V* curves, the current response of the cells is simulated by given a set of the potential drops across the active layers. The potential drops are the boundary conditions for Poisson's equation (**Figure** **3**a). At the steady state, a voltage is applied to cells with *V*_appl_, and the potential drop across the active layer becomes

(18) $$V=V_{\mathrm{appl}}-V_{\mathrm{bi}}$$

The work functions for the anode and the cathode determine the built‐in potential, which is read as

(19) $$V_{\mathrm{bi}}=\frac{1}{e}\left(E_{f1}-E_{f2}\right)$$

where *E*_f1_ and *E*_f2_ are work functions for the anode and the cathode, respectively.

*Transient Problems*: Nanosecond and microsecond transient current measurements have also been applied to characterize the thin film devices. In transient problems, the transient photovoltage or photocurrent response is detected after an external disturbance, such as a short light pulse (Figure 3b). In modeling the transient problems, a closed circuit is considered.^68^, ^70^ The closed circuit has been considered by containing the voltage source, *V*_s_; an effective resistance, *R*; and an organic device with the voltage drop defined as *V*_app_, the voltage drop at the resistance is

$$V_{R}(t)=V_{s}-V_{\mathrm{appl}}(t),$$

By considering the closed circuit, Poisson's equation is replaced by

(20) $$\varepsilon \frac{\partial E(x,t)}{\partial t}=\frac{V_{\mathrm{app}}-V_{\mathrm{bi}}+\int_{0}^{L}E(x,t)dx}{SR}-j(x,t)$$

Equation (20) contains boundary conditions and has a partial derivative form of time. Its function is equivalent to Poisson's equation. It has the advantage of simulating the transient problem, because the voltage drop at the resistance well defines the transient photocurrent and the transient photocurrent.

### 2.3 The Simulation Methods and Solar Cell Simulators

**Figure 4:**
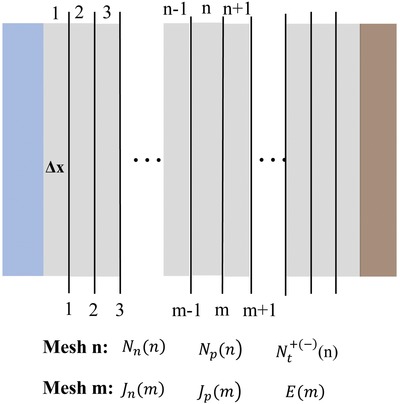
Schematic on the discretization of the device into binary meshes (m, n). Charge carrier density, traps are defined at the mesh (n); current density and electric filed are defined at the meshes (m).

The finite difference numerical method is used to solve the coupled nonlinear equations. The first step is to discretize the device into spatial meshes. Typically, binary grids are merged with (*m*) meshes that were specified for spatial derivatives and (*n*) meshes that were specified for temporal derivatives.^10^, ^19^, ^70^, ^71^ In other words, the charge carrier density and trap density are defined on (*n*) meshes, while the current density and electric field are defined on (*m*) meshes. By the above method, all the spatial derivatives are replaced by differences between the grids (**Figure** **4**).

When calculating the electron current density *J_n_*(*m*), the electron density *N_n_*(*n*), *N_n_*(*n* − 1) of the nearest point needs to be included. However, Scharfetter and Gummel have demonstrated that if an averaged electron density of *N_n_*(*n*), *N_n_*(*n* − 1) is used, the rapid carrier density variance between meshes triggers numerical instability.^72^ They solved the problem by proposing an exponential variation of the carrier density with the meshes grid, while the fixed electric field and current density tend to be constant. In this procedure, the equation is integrated analytically, and its finite difference form (take electron for example) changes into a form as follows

(21) $$\frac{dN_{n}\left(n\right)}{dt}=\frac{J_{n}\left(m+1\right)-J_{n}\left(m\right)}{q\Delta x}+G\left(m\right)-R\left(m\right)$$

And the equilibrium form

$$\frac{J_{n}\left(m+1\right)-J_{n}\left(m\right)}{q\Delta x}+G\left(m\right)-R\left(m\right)=0$$

(22) $$J_{n}\left(m\right)=\frac{q\mu_{n}k_{B}TE\left(m\right)}{1-\exp\left(-E\left(m\right)\Delta x\right)}\times \left[N_{n}\left(n\right)-N_{n}\left(n-1\right)\exp\left(-E\left(m\right)\Delta x\right)\right]$$

The procedure effectively circumvents the instability, and it was followed by many of the device model simulators.^2^, ^19^, ^70^, ^71^

Having formulated the model equations in the above form, the next problem is how to implement the numerical solution. Several algorithms or programs are used to address the equations that are expressed above. Gear's method, MATLAB's ode15s, and the Runge–Kutta^71^ method are adapted to integrate forward in time, and Fourier's method, Newton–Raphson iteration algorithms,^66^ and Newton and Gummel^73^ methods are used to solve the equilibrium forms.

In addition to self‐developed simulation codes, several solar cell simulators are available to the PV community. Recently, some of them have been employed to simulate normal OSCs and planar PSCs, such as Analysis of Microelectronic and Photonic Structures‐1D (AMPS‐1D),^61^ Solar Cell Capacitance Simulator (SCAPS),^73^ and AFORS‐HET.^66^ Although these simulators are developed in the simulation amorphous silicon, heterojunction silicon, copper indium gallium selenide(CIGS) solar cell, Cu2ZnSnS4(CZTS)‐based solar cells, and cadmium telluride (CdTe) solar cells, they are believed to be very general solar cell simulation programs, which numerically solve the semiconductor drift‐diffusion device equations and Poisson's equations. To better treat the carrier transport at interfaces, thermionic emission and specific tunneling mechanisms are adopted in most of the simulators.^65^, ^73^ Most of the simulators extend the simulation ability to simulate an arbitrary sequence of semiconducting layers and interfaces with defects that are distributed within the hand gaps. The recombination is modeled by Auger, direct band‐to‐band, and Shockley–Read–Hall recombination.^2^, ^66^, ^73^

## 3 The Application of the Device Model Simulation to Organic Devices

### 3.1 Organic Devices

#### 3.1.1 Single Layer OSCs

The Au/ZnPc [zinc‐phthalocyanine]/Al device configuration is one of the earliest single layer small molecule OSCs.^74^ Later, conjugated polymers, such as PPV and its derivatives, have been introduced into the solar cells with a structure such as ITO/MEH‐PPV/Ca, for example.^24^ In single‐layer solar cells, the organic semiconductors are sandwiched between two different metals. Typically, high work function metal, such as indium tin oxide (ITO), is used as the anode. Low work function metal, such as Al, Ca, Ag, or Mg, is a cathode metal. Usually, the adaption of the metal oxide between the organic layer and the metal electrode has been reported to enhance the efficiency and the stability.

**Figure 5:**
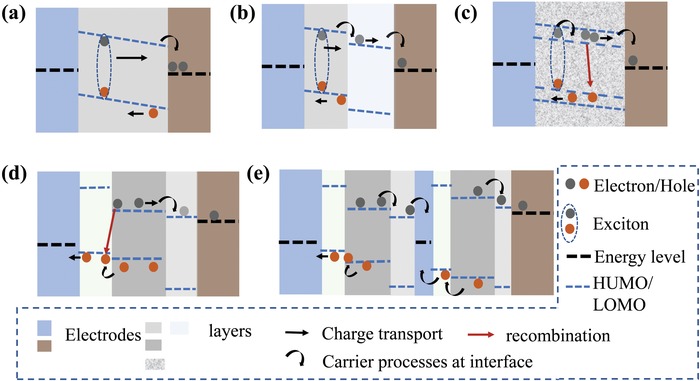
A schematic on the thin film devices and the underlying physical processes. a) The simple device consisting of a single organic layer between metal contacts. After absorbing photons, excitons generate and diffuse toward the contact where they dissociate to yield charge pairs. b) Bilayer donor–acceptor heterojunction. After reaching the donor–acceptor interface, exciton dissociates, leaving an electron on the acceptor. c) Bulk‐heterojunction with well‐blended donor and acceptor layer. The donor and acceptor exciton dissociation interface distributes with a dispersive manner. d) Charge carrier recombination at the interface between layers is thought to be the main energy loss. e) Perovskite‐perovskite tandems integrating sub cell with a low *E*_g_ perovskite layer and sub cell with a high *E*_g_ perovskite layer.

The device physics of single‐layer OSCs are divided into the processes as follows (**Figure** **5**a).^75^ After absorbing photons, an electron is excited from HOMO to LUMO, and a tightly binding electron–hole pair (exciton) is formed. However, excitons cannot dissociate spontaneously to free charge carriers, for the dissociation of exciton requires additional energy of ≈100 meV or an electronic structure with energy level discontinuity. In single‐layer organic devices, the metal/organic interface exhibits the electronic structure that assists the dissociation of the excitons. After excitation, excitons should diffuse to the metal/organic interface and then dissociate by injecting an electron into the metal and leaving holes in the semiconductors. The hole charge carriers move under the action of the electric field, some of which are eventually collected by electrodes, and some of which recombine. At the beginning of modeling the thin film device, to reproduce the current was the primary focus. In addition, the short exciton diffusion length, surface charge recombination, traps, and inefficient charge carrier collection are the main limitations of single‐layer OSCs. Single layer solar cells of this type were reported to have a low PCE of 0.01–1%.^76^, ^77^

#### 3.1.2 Bilayer OSCs

The first bilayer thin film OSC has a structure denoted by ITO/In_2_O_3_/CuPc/PV/Ag, which was invented by C. W. Tang in 1986.^78^ The copper phthalocyanine layer, which is ≈30 nm thick, was deposited by conventional vacuum evaporation on the indium tin oxide (ITO)‐coated glass, which provided a transparent conducting substrate. The perylene tetracarboxylic derivative was ≈50 nm thick. In addition to the small molecule OSCs, conducting polymer/C_60_ cells were also commonly studied bilayer OSCs, such as the C_60_/MEH‐PPV/ITO configuration in Figure 1a. In the configuration, MEH‐PPV was first spin‐coated onto the ITO‐coated glass substrate, and then the fullerene was vacuum evaporated onto a MEH‐PPV layer.^79^

In the bilayer device, an electron donor and an electron acceptor material are stacked together with a planar interface. The bilayer OSCs differs from conventional single‐layer cells in that the interface between two thin organic layers is crucial for determining its photovoltaic properties. Notably, as shown in Figure 5b, excitons dissociate at the donor and acceptor interface rather than the interface between the metal and semiconductor layers. In addition, the interface works as the charge blocking layers by holding an electron at one side, while the hole at the other side, the electron and the holes can only transverse across the interface by recombination or field‐assisted injection (large electric field). By introducing a donor/acceptor interface, the exciton dissociation and charge collection are enhanced in the bilayer structures. The polymer‐fullerene bilayer heterojunction device attained 3.6% PCE.^80^

#### 3.1.3 Bulk‐Heterojunction OSCs

In single‐layer and bilayer OSCs, most of the excitons recombine before reaching the dissociating region, which leads to a low exciton dissociation efficiency. Recognizing this fact, the bulk heterojunction concept has been introduced to improve the exciton dissociation in the OSCs. In bulk heterojunctions, the polymers or molecules are blended (Figure 1b). Thus, the donor–acceptor microstructure spread to small regions within the exciton diffusion length. In an ideal bulk heterojunction, as shown in Figure 5c, all excitons will diffuse into the donor–acceptor interface during the lifetime, and the free charge generation rate only depends on the physics of exciton dissociation. Moreover, the bulk heterojunction requires percolation pathways for the hole and electron to transport toward contacts. In other words, the donor and acceptor phases form an interpenetrating network. Thus, the bulk heterojunction devices are much more sensitive to nanoscale morphology in the blend. Among many of the bulk heterojunction devices, polymer/fullerene OSCs, including the RRP_3_HT/PCBM blend,^81^ the MDMO‐PPV(poly[2‐methoxy‐5‐(3,7‐dimethyloctyloxy)]‐1,4‐phenylenevinylene)/PCBM blend, and the P_3_HT(poly(3‐hexylthiophene‐2,5‐diyl)/PCBM blend were intensively studied, and a PCE above 5% PCE under AM 1.5 was achieved.^82^

### 3.2 The Device Model Analysis on the Current of the Thin Film Devices

#### 3.2.1 Space‐Charge‐Limited Current and Injection‐Limited Current

For the thin film device, the ability to inject charge at the boundary determines the concentration of the space charge, which ultimately affects the current characteristics of the device. Therefore, charge injection barriers can be used to distinguish the contact conditions. For charge injection barriers less than ≈0.3 eV, the charge can be efficiently injected into the device. The contact is an ohmic contact. Under the ohmic contact condition, the current flow is space charge limited in typical organic device parameters, such as the device length and the charge carrier mobility. For a charge carrier injection barrier larger than 0.3 eV, there is not enough injected charge at the interface. The contact is a nonohmic contact, and the current flow is injection limited. Under an ohmic contact condition, the typical *J*–*V* curves are limited by space charge‐induced screening on the electric field in the semiconductor material. Lampert modeled the current, which was mainly injected in a plane parallel structure, by solving the coupled Poisson's and drift‐diffusion equations approximately.^16^, ^69^ The flow of the electrons, which are injected by one ohmic contact between metal and an insulator, is studied, and a space‐charge‐limited‐current is the result with a quadratic function of voltage. The analytical solution of drift‐diffusion equations is limited in the analysis of complex devices. However, the reproducing the *J*–*V* curves of the single‐layer diodes from the device physics model is the basis for detailed understanding of the thin film devices, including thin film solar cells and thin film light emitting diodes.

**Figure 6:**
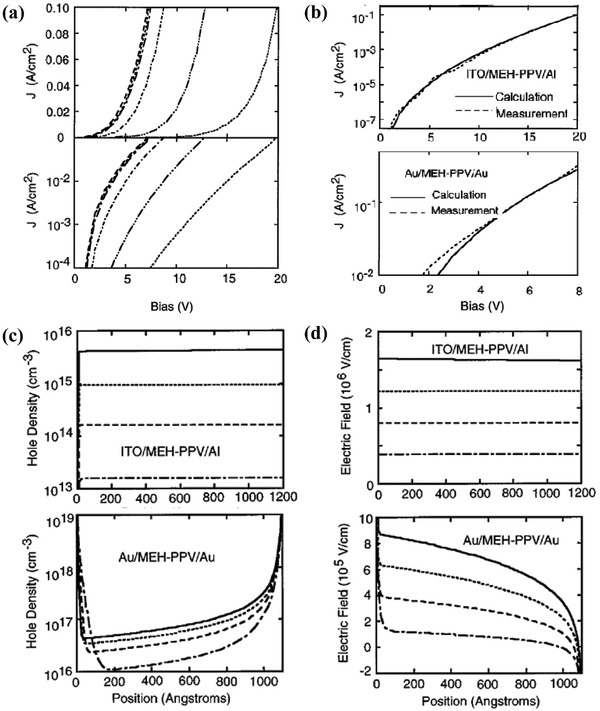
a) The calculated results for 0.1, 0.2, and 0.3 eV barriers are nearly the same. For these cases, the current flow is space charge limited. As the energy barrier is further increased, the current is decreased, indicating that the current flow becomes injection limited. b) The barrier for electron injection to MEH‐PPV from Al is about 1.4 eV and for hole injection from ITO about 0.6 eV. The barrier for hole injection from Au into MEH‐PPV is about 0.1 eV. c) Calculated hole density as a function of position for the Al/MEH‐PPV/ITO device in the upper panel, and the Au/MEH‐PPV/Au device lower panel. In the upper panel, the bias voltages are 20 V (solid line), 15 V (dotted line), 10 V (dashed line), and 5 V (dotted line). In the lower panel, the bias voltages are 8 V (solid line), 6 V (dotted line), 4 V (dashed line), and 2 V (dot‐dashed line). d) The calculated electric field as a function of position for the Al/MEH‐PPV/ITO device (upper panel) and the Au/MEH‐PPV/Au device (lower panel). Reproduced with permission.^9^ Copyright 1997, AIP Publishing.

The computer‐aided solution of the device model improves the accuracy of the current analysis for complex devices. The numerical simulation enables the device model to accurately fit the experimental *J*–*V* data and obtain more detailed information, such as electric field distribution, and charge carrier density. Detailed information has a clear guiding role for the dominant physical mechanism, which is helpful for the device design. For example, Smith and co‐workers showed the space‐charge‐limited current, and the injection‐limited current in the same model by numerically solving the device model systematically.^9^ The device model was coupled with the current at an interface described as Equation (13). The results show that for energy barriers less than ≈0.3 eV, the current is nearly the same, the current flow is space charge limited (**Figure** **6**a), and the electric field in the structure is highly nonuniform (Figure 6d). For larger energy barriers, the current injection is limited. In the case of injection limitation, the net injection charge is relatively small, the electric field is almost uniform, and the space charge effect is not important.

The calculation gives a good description of the measured *J*–*V* characteristics over a wide current range in this injection‐limited situation for the Al/MEH‐PPV/ITO device, and in the space‐charge‐limited situation for the Au/MEH‐PPV/Au device. For an MEH‐PPV diode with ITO hole injecting contacts and Al electron injecting contacts, the injection barrier for the electron from MEH‐PPV to Al is ≈1.4 eV, and the injection barrier for the hole is ≈0.6 eV. The calculation gives a good description of the measured *J*–*V* characteristics over a wide current range in the injection‐limited situation, as shown in the upper panel of Figure 6b. For the MEH‐PPV diode with ITO‐hole‐injecting contacts and Au‐electron‐injecting contacts, the injection barrier for the hole from MEH‐PPV to Au is ≈0.1 eV. With such small injection barriers, the current is space charge limited. The hole density and electric field are strongly varying functions of the position, as shown in the lower panel of Figure 6c,d. The calculation has given a reasonable description for the measured *J*–*V* characteristics in both the space‐charge‐limited and injection‐limited situations.

#### 3.2.2 Space‐Charge‐Limited Current in Organic Solar Cell

**Figure 7:**
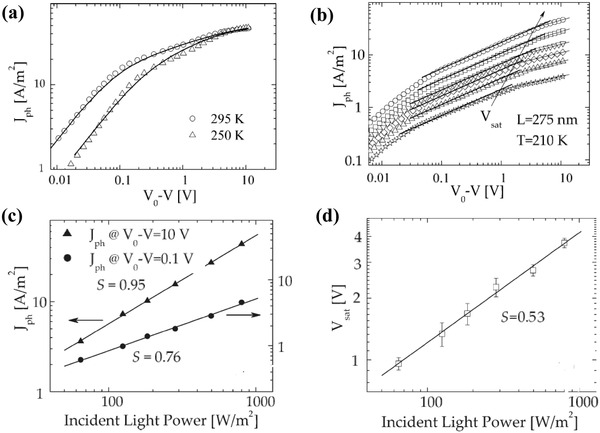
a) Effective photocurrent (*J*_ph_ = *J*_L_ − *J*_D_) as a function of applied voltage (*V*_0_–*V*) of MDMO‐PPV: PCBM 20:80 device (symbols), at 295 and 250 K. The solid line represents the numerical calculation including diffusion, field dependent of generation rate G(T, E) and recombination, for a device with a thickness of 120 nm. Reproduced with permission.^19^ Copyright 2007, WILEY‐VCH Verlag GmbH & Co. b) Incident light power dependence of the photocurrent (*J*_ph_) versus the effective voltage (*V*_0_–*V*) measured at *T* = 210 K. The solid (thick) line represents the calculated *J*_ph_ from the space charge limited current theory. The arrow indicates the voltage at which *J*_ph_ shows the transition to the saturation regime. c) Incident light power dependence of the photocurrent *J*_ph_ at an effective voltage of *V*_0_ − *V* = 0.1 V and *V*_0_ − *V* = 10 V (symbols). d) Saturation voltage versus Incident light power. The slope (*S*) determined from the linear fit (solid lines) to the experimental data is written on the figure. Reproduced with permission.^20^ Copyright 2005, American Physical Society.

If the photoinject charges cannot be effectively collected, space charge accumulation will also occur. The charge accumulation arises in the solar cells with an organic semiconductor of low charge carrier mobility. The imbalance between the electron and the hole charge carrier mobility, e.g., electron mobility in the semiconductor, is a factor of 100 larger than the hole mobility and can cause space charge accumulation, resulting in a space‐charge‐limited current in OSCs. This kind of space‐charge‐limited current often happens in OSCs, in that the organic semiconductor has an electron mobility order higher than the hole mobility. Goodman and Rose have given one approximate theory of the double extraction of charge carriers from a photoconductor layer. In their calculation, for regimes of the current versus the applied voltage behavior are predicted with *I* ∝ *V* at a low voltage; a transition region between *I* ∝ *V* and *I* ∝ $\sqrt{V}$ at a higher voltage; *I* ∝ $\sqrt{V}$ at still a higher voltage, and a saturation value at a very high voltage.^83^ Blom et al. has applied the device model simulation to analyze the current–voltage characteristics of the polymer: Fullerene bulk heterojunction solar cells.^19^ The transport of electrons/holes, the space charge, and the carrier mobilities were found to be closely connected with typical *J*–*V* curves. In the MDMO‐PPV: PCBM 20:80 device (**Figure** **7**a), with a mobility difference of only a factor of ten, the overall carrier densities are rather low; the space‐charge effects only play a minor role, leading to a nearly constant field in the device. For low effective voltages, *V*_0_–*V*, the photocurrent increases linearly with the effective voltage due to direct competition between diffusion and drift currents, as predict by Goodman and Rose. At a higher effective voltage, all the free charge carriers are extracted for zero recombination. However, in BEH_1_BMB_3_‐PPV:PCBM, the electron mobility in the PCBM phase is a factor of 125 larger than the hole mobility. The experimental *J*_ph_ clearly shows a square root dependence on voltage in the voltage region above 0.06 V, as predicted by the space‐charge‐limited current in the solar cells (Figure 7b). In a double logarithmic plot, the experimental *J*_ph_ is a function of incident light power for two different voltages, at *V*_0_ − *V* = 0.1 V in the square root regime and at *V*_0_ − *V* = 10 V in the saturation regime. The slope S determined from the linear fit (solid lines) to the experimental data amounts to *S* = 0.76 in the square root part and *S* = 0.95 in the saturation part at high voltages, as shown in Figure 7c. Figure 7d shows the one‐half power fitting of the saturation voltage varies as the light intensity. The 1/2 power dependence of *J*_ph_ on the voltage and 3/4 dependence on incident light power is a strong indication of the occurrence of a space‐charge‐limited photocurrent of the understudied device.

#### 3.2.3 Trap Effects on the Current Density

The trap states have a significant influence on the charge transport through the device. Some phenomenological current–voltage curves show characteristics that are related to the traps in the semiconductor. By modeling the phenomenological relations, detailed information about the trap states can be revealed. The trap state density, trap state energy distribution, and trap state characteristic energy are important information with which to characterize the trap states. Mark and Helfrich modeled the current that is affected by exponentially distributed traps, and they predicate that the quadratic function of voltage changes into the following form

(23) $$j\propto \frac{{\left(V_{a}-V_{\mathrm{bi}}\right)}^{l+1}}{d^{2l+1}}$$

**Figure 8:**
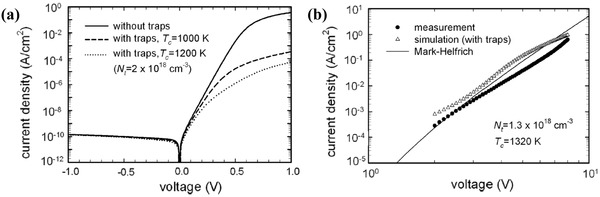
a) Simulation of the effect of an exponential distribution of traps on the *I*–*V* characteristics of an organic diode. The traps result in a deviation of the forward current at low biases from the exponential growth, which can be interpreted in terms of an ideality factor. Ideality factor becomes higher with increasing a characteristic temperature *T*_c_. b) *J*–*V* data in the bulk‐limited regime (2 to 8 V) plotted in log–log scale together with the best‐fit simulation and the prediction of Mark–Helfrich's model (trap‐limited SCLC). Reproduced with permission.^8^ Copyright 2011, AIP Publishing.

Traditionally, the current is plotted in log–log coordinates, and the slope of the straight line (*l* + 1) was used as direct access to the characteristic temperature (*T*_C_) of the trap distribution.^84^ Kim et al. studied the physics of single‐layer organic diodes with traps by the device model method.^8^ The effect of an exponential distribution of traps on the *J*–*V* characteristics of an organic diode has been simulated by the device model method. At low biases (0–1 eV), the traps result in a deviation of the forward current from exponential growth, which can be interpreted in terms of an ideality factor. The influence of traps on the *J*–*V* characteristics of the organic diode is shown in **Figure** **8**a and is discussed in Equation (24), where *n* = 1/(1 − α) is the ideality factor. There are many studies on the ideality factors in single‐layer diodes with various organic semiconductors, and the measured ideality factors ranged from 1.6 to 4.3.^85^ Kim's simulation work attributed the ideality factor to the effects of traps

(24) $$j=j_{s}e^{qV_{a}/nkt}$$

In the bulk‐limited regime (2 to 8 V), Figure 8b shows the measured and simulated *J*–*V* data in the log–log scale. The reasonable linearity of the measured curve ascertains a power‐law relationship between the current and the voltage. The curve was calculated with the Mark–Helfrich model (Equation (23)) with the extracted trap density *N*_t_, and a characteristic temperature *T*_c_ is plotted in Figure 8b, as well. The estimated slope of the curve is 5.4, and the simulation reveals that the current is strongly limited by traps.

#### 3.2.4 Modeling of the Transient Current

**Figure 9:**
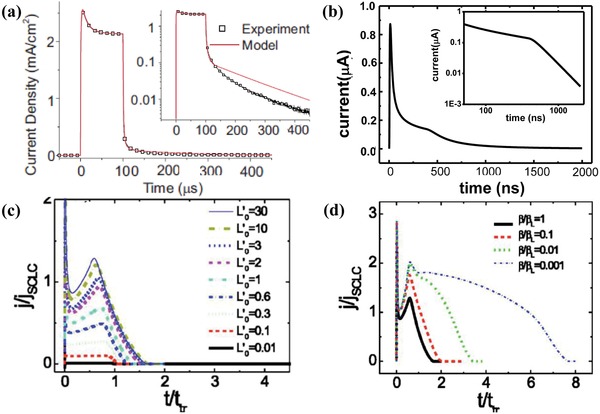
The transient photocurrent response of devices. a) Comparison of the experimental (squares) and the calculated (line) microsecond transient photocurrent response after a light pulse. The inset displays these curves on a log‐linear plot highlighting the deviation between experiment and model at low photocurrent densities after turning off. The transient peak can be explained by the buildup of trapped electrons, and detrapping rate constant used of 2.2 × 10^5^ s^−1^ qualitatively reproduces the photocurrent tail after turning off the light pulse. Reproduced with permission.^86^ Copyright 2009, AIP Publishing. b) The simulated nanosecond transient photocurrent response of the device with traps after a light pulse. The inset displays the current on an in log–log scale, the hyperbolic shape is the result by the dispersion of the traveling charge packet in disorder material. c) Transient current as a function of light intensity. The numerically calculated transient photocurrent response with the intensity given in normalized units, *t*_tr_ = *d* ^2^/*µU*_0_. d) the numerically calculated transient current for various bimolecular recombination rate β/*βL* ratios. Reproduced with permission.^87^ Copyright 2005, American Physical Society.

The device model simulation of the transient current response has revealed more details about the physics of the thin film devices. Much information about carrier dynamics can be obtained from transient photoelectric characteristics. It is helpful to study the properties of semiconductor materials and devices. The Langevin bimolecular recombination coefficient, trapping/detrapping rate constant, and charge carrier mobilities have been extracted by modeling the transient photocurrent and the transient photovoltage. Greenham and co‐workers have simulated the microsecond transient photocurrent responses by illuminating the devices with square pulses of light.^86^ The result presents the results of time‐dependent drift‐diffusion modeling with traps, as shown in Equations (5), (6), (7), with the model reproducing a transient peak multiexponential decay. The simulation revealed that the transient peak can be explained by the buildup of trapped electrons near the anode. In addition, the detrapping rate constant of 2.2 × 10^5^ s^−1^ qualitatively reproduces the photocurrent tail after turn off, which confirms that this tail originates from charges that are slowly detrapped as shown in **Figure** **9**a. Li et al. used the drift‐diffusion current with the trap by Equation (9) to study the trap effect on charge carrier transport at the nanosecond.^71^ They predicted well the hyperbolic shape of the empirically observed photocurrents in disordered materials, as was described by the expression

(25) $$i\left(t\right)\approx \{\begin{array}{c} t^{-\left(1-\alpha_{1}\right)},t>t_{t}\\ t^{-\left(1-\alpha_{2}\right)},t<t_{t} \end{array}$$

where α_1_ and α_2_ are the dispersion parameters at short and longer times, respectively, as shown in Figure 9b.^89^ Pivrikas et al. applied the charge carrier extraction mechanism in high light intensity space‐charge‐limited transient current to investigate the bimolecular recombination at the poly(3‐hexylthiophene) film.^87^ Under the space‐charge‐limited condition, the external electric field in the photogenerated charge carrier reservoir is screened within a shorter time scale than the recombination time, and the transient curves of the photocurrent are dependent on the recombination. They assumed that the bimolecular recombination at the layers were

(26) $$\frac{dp}{dt}=-\beta np=-\beta p^{2}$$

They measured the time‐of‐flight transient photocurrent responses and fitted the photocurrent numerically with various γ = β/β_L_ ratios. The magnitude of the Langevin bimolecular recombination coefficient can then be calculated by using the following relation

(27) $$\beta_{L}=\frac{e\left(\mu_{p}+\mu_{n}\right)}{\varepsilon_{0}\varepsilon_{r}}$$

where *µ*_p_(*µ*_n_) is the mobility of holes (electrons).^88^ Figure 9c shows the transient photocurrent responses dependence on light intensity in the time‐of‐flight measurements. At low light intensity, the transient photocurrent responses clearly showed a typical plateau and a drop in the current level as the carriers reach the opposite electrode. The current drops at the current transit time *T*_tr_, from which the charge carrier mobility is calculated based on *t*_tr_ = *d* ^2^/*µU*_0_. At intermediate light intensities, they saw the development of a cusp, and at high light intensities, the transient photocurrent responses saturate as a function of the light intensity and show a well‐developed space charge transient current cusp. The abundant transient current characteristics are related to the carrier motion inside the device. The numerically simulated time‐of‐flight transient photocurrent responses for various γ ratios are presented in Figure 9d. It shows the dependence of the transient photocurrent responses on the bimolecular recombination rate for high light intensities. For fast bimolecular recombination (γ = 1), the transient photocurrent responses show space charge behaviors, with the extraction time being shorter than the carrier transit time. The numerically calculated transient photocurrent responses with γ = 1 fit the measured data well. Thus, they argued that the bimolecular recombination coefficient in RRaPHT semiconductors is in accordance with what is expected from Langevin recombination.

### 3.3 The Device Model Analysis on the Charge Carrier Dynamics At Interfaces

#### 3.3.1 Thermionic Current and Tunneling Current at the Interface

Interfaces between the layers of the thin film device have a great influence on the thin device performance. Charge carrier thermionic emission and quantum tunneling, such as intraband tunneling and trap‐assisted tunneling, are the possible charge carrier processes at the interface. Exploring the rules of these processes and their effects on the performance of the device is an important issue in the research of thin film devices. The theory of the thermionic current is given in Section 2.2.3, and the theory for the tunneling current at the metal/organic interface are included in the device model by Smith. Herein, we give a brief review of the thermionic current and the tunneling current at the interface.

**Figure 10:**
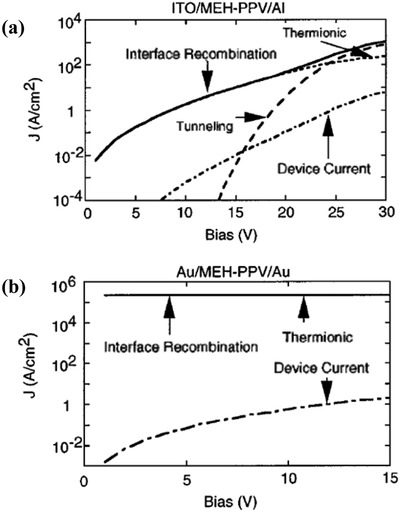
Calculated the injection current components and the total device current as a function of bias for the Al/MEH‐PPV/ITO device (a) and the Au/MEH‐PPV/Au device (b). The solid line is the current due to interface recombination, the dotted line is the current due to thermionic emission, the dashed line is the current due to tunneling, and the dot‐dashed line is total device current. For the device in (b) panel, there is no tunneling current, and the interface recombination and thermionic emission currents are so close that they cannot be distinguished in the figure. Reproduced with permission.^9^ Copyright 1997, AIP Publishing.

It is believed that the tunneling current through an interface barrier is non‐negligible when the barrier is thin (say <3 nm) under a high electric field condition and that the effective mass for tunneling is low. The tunneling current more likely happens in inorganic solar cells with a high doping density. The simulated current at the metal/organic interface by Smith and co‐workers could serve as an example. The thermionic injection current, the collection current that is the time‐reversed process of the thermionic injection process, and the tunneling current were studied by the device model in their simulations.^9^ They calculated values of the injection current components and the total device current as a function of bias for the Al/MEH‐PPV/ITO device and the Au/MEH‐PPV/Au device. In all cases, as shown in **Figure** **10**, Smith et al. found that the backflowing interface recombination current very nearly cancels the sum of the injection currents, which means that the thin film device works at the quasi‐equilibrium conditions regardless of whether the unequilibrium charges are thermally injected in the transistor or are photoinjected in the solar cells. This confirms our previous analysis of the equivalence of the current density boundary and the charge carrier density boundary in Section 2.2.4.

Moreover, the tunneling current only takes a considerable role at a high bias above 15 V in thin film devices. It appears reasonable that tunneling currents are difficult to generate in solar cells, most of which operate at a bias ≈1 V. However, it should be taken seriously when the electric field distribution inside the device is nonuniform or there are low dimensional structures.

#### 3.3.2 The Physical Processes of Exciton at the Interface

After absorbing photons, tightly binding electron–hole pairs (excitons or polaron pairs) are excited in organic materials. To generate the photocurrent, excitons should first dissociate into free electrons and holes. However, excitons cannot dissociate spontaneously, for the dissociation of exciton requires additional energy or an electronic structure with energy level discontinuity. The donor–acceptor interface of bilayer OSCs, the donor–acceptor structure in bulk heterojunction OSCs, and a sufficiently strong electric field can promote exciton dissociation. In bilayer devices, the donor–acceptor interface gives an energy level discontinuity. Exciton is diffused to the interface and is dissociated by keeping electrons at acceptors, while holes are kept at donors. The devices show significant improvements in PCE over single‐layer devices. However, most of the excitons recombine before reaching the dissociating region, which leads to low exciton dissociation efficiency. The donor–acceptor structure in the bulk heterojunction design is within the reach of excitons and has been introduced to improve the exciton dissociation in the OSCs. In addition to energy level discontinuity, the electric field is favorable in exciton dissociation by pulling the electron–hole pairs apart, which results in dependence of the free charge carrier generation rate on the applied voltage.

The device model method has been used to model the bilayer device and bulk heterojunction devices with detailed physics of exciton dissociation. Typical photocurrent characteristics have been proven to be caused by the exciton dissociation. Barker et al. included the polaron pairs dynamics model at the polymer–polymer interface in bilayer polymer photovoltaic devices.^2^ The polaron pairs may either recombine monomolecularly with a coefficient *k*_rec_ corresponding to their lifetime τ_rec_, or dissociate into free charges with a field‐dependent coefficient *k*_diss_ (*E*). The density of the polaron pairs at interface *X*, therefore, follows the rate equation as

(28) $$\frac{\partial X}{\partial t}=G_{X}-k_{\mathrm{rec}}X-k_{\mathrm{diss}}\left(E\right)X+F_{X}$$

In the above equation, the polaron pair formation rate *F_X_* and the field‐dependent coefficient *k*_diss_ (*E*) must be specified. They developed a polaron pair dissociation theory. The dissociative pairs are those that are only able to escape the mutual coulomb potential over half a surface. The dissociate across the section is

(29) $$k\left(0\right)=\int_{0}^{2\pi }d\psi \int_{0}^{\pi /2}\sin\theta d\theta \exp\left(-\frac{U_{B}}{k_{B}T}\right)$$

where *U*_B_ is the polaron pair binding energy, and *A* is a constant related to the attempt frequency for escape. Under the negative applied field, the carrier escape barrier is lowered, and the dissociation of the exciton is enhanced. The dissociation coefficient is given as follows

$$k_{\mathrm{diss}}\left(E\right)=\frac{k_{\mathrm{diss}}\left(0\right)}{M}\left[\exp\left[M\right]\left(1-\frac{1}{M}\right)+\frac{1}{M}\right]$$

(30) $$M=\frac{e}{k_{B}T}\sqrt{\frac{-eE}{\pi \varepsilon_{0}\varepsilon_{r}}}$$

At positive applied fields, polaron dissociation will be suppressed, and the dissociated coefficient is given as

(31) $$k_{\mathrm{diss}}\left(E\right)=k_{\mathrm{diss}}\left(0\right)\frac{4\pi \varepsilon_{0}\varepsilon_{r}{\left(k_{B}T\right)}^{2}}{e^{3}E}\left(1-\exp\frac{e^{3}E}{4\pi \varepsilon_{0}\varepsilon_{r}{\left(k_{B}T\right)}^{2}}\right)$$

The formation of polaron pairs on opposite sides of the polymer–polymer interface by the bimolecular capture of free charges. The polaron pair formation rate is given by

(32) $$F_{X}=\frac{nph\left(\mu_{n0}+\mu_{p0}\right)e}{3\varepsilon_{0}\varepsilon_{r}}$$

where *n* is the electron density in the electron transporting layer at the interface, and *p* is the hole density in the hole transporting layer at the interface.

**Figure 11:**
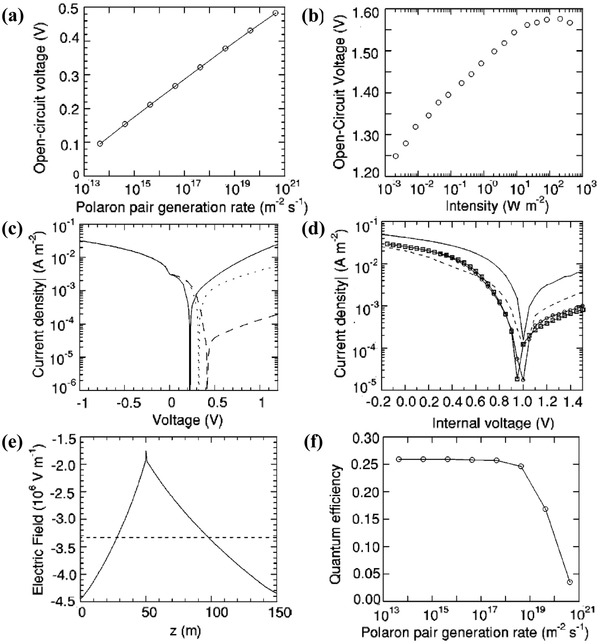
The modeled and measured bilayer organic devices with exciton dissociation at the donor–acceptor interface. a) Open‐circuit voltage versus polaron pair generation rate with a lifetime τ_rec_ = 1 µs. A logarithmic dependence of *V*_OC_ on intensity is reproduced over a wide range of intensities. b) Measured open‐circuit voltage for an ITO/PFB(50 nm)/F8BT(100 nm)/Al device as a function of incident intensity at 458 nm. The data shows a logarithmic dependence of *V*_OC_ on intensity. c) Modeled current–voltage curves with τ_rec_ = 1 µs and a polaron pair generation rate of 4.3 × 10^17^ m^−2^ s^−2^. The anode barrier is 0.5 eV and the cathode barrier is 0.4 eV (solid line), 0.5 eV (dotted line), or 0.6 eV (dashed line). d) Measured current density versus internal voltage for ITO/PFB(50 nm)/F_8_BT(100 nm) cathode devices, under illumination at 459 nm with an intensity of 7 W m^−2^. The cathodes are gold (circles), copper (squares), chromium (solid) line, and aluminum (dotted) line. e) Modeled electric field versus position at −0.5 V with τ_rec_ = 1 µs, at polaron pair generation rates of 4.3 × 10^17^ m^−2^ s^−2^(dashed line) and 4.33 × 10^19^ m^−2^ s^−2^ (solid line). f) Quantum efficiency versus polaron pair generation rate at −0.5 V with τ_rec_ = 1 µs. Reproduced with permission.^2^ Copyright 2004, American Physical Society.

Barker et al. revealed the *J*–*V* characteristics of the bilayer device by the device model simulation by considering the field‐dependent polaron pair dissociation rate. **Figure** **11**a,b shows the modeled and measured light intensity dependence of *V*_OC_. High light intensity corresponds to a high polaron pair yield rate. The experimentally observed logarithmic dependence of *V*_OC_ on the intensity is reproduced over a wide range of intensities. Figure 11c,d shows the calculated and measured *J*–*V* curves. The calculated current–voltage characteristic for a device reproduces many essential features in the experimental curves, i.e., a linear increase in the photocurrent with increasing negative voltage, whereby the quantum efficiency increases from 0.05 at 0 V to 0.46 at −1 V. Figure 11e,f gives the modeled electric field versus the position and quantum efficiency at different polaron pair generation rates. The results reveal that short‐circuit quantum efficiency is determined by the competition between the polaron pair dissociation and recombination. At low light intensity, the electric field is a constant through the device, and the drift current dominates the interface. At high light intensities, however, space charge in the device significantly reduces the electric field at the interface, i.e., the field becomes less negative, thus leading to reduced polaron pair dissociation and short‐circuit quantum efficiency.

**Figure 12:**
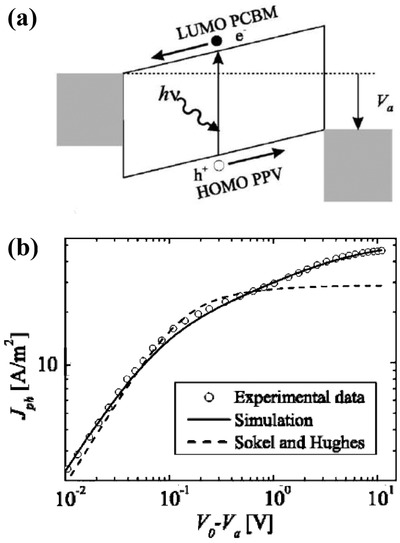
a) The schematic on the charge carrier generation, and b) the device model fitting of the photocurrent density. The linear behavior at low effective voltage is attributed to the competition between diffusion and drift currents. At higher effective voltage, the current is not saturated because of the field dependence of the generation rate. Reproduced with permission.^1^ Copyright 2005, American Physical Society.

Onsage and Braun have given similar exciton dissociation equations, and Blom and co‐workers applied it to simulate the bulk heterojunction devices.^1^, ^18^, ^90^ In their formulas, exciton dissociates into coulomb‐bonding electron–hole pairs, and the free carriers are obtained through electric field assisted dissociation of electron–hole pairs. The dissociation coefficient *k*_dis_ is given by Braun's model by assuming an electron–hole pair with a separated distance *a*, and

(33) $$k_{\mathrm{diss}}\left(E\right)=\frac{3R}{4\pi a^{3}}e^{-\Delta E/k_{B}T}J_{1}\left(2\sqrt{-2b}\right)/\sqrt{-2b}$$

where, Δ*E* is the binding energy, *b* = *qE*/(8*πεk* ^2^ *T* ^2^), E is the electric field strength, and *J*_1_ is the Bessel function of order 1. The charge carrier generation and the following charge transportation are shown by the schematic of **Figure** **12**a.

They have modeled the *J*–*V* curves of the OC_1_C_10_‐PPV/PCBM devices with exciton dissociation at the donor–acceptor interface described by Equation (33).^1^ The effective photocurrent density *J*_ph_, (Figure 12b) which is obtained by subtracting the dark current from the current under illumination, is plotted as a function of the effective applied voltage *V*_0_–*V*_a_. The linear behavior at low effective voltage is the result of direct competition between the diffusion and drift currents. At higher effective voltage, all free charge carriers are extracted for zero recombination, and the photocurrent saturates to the generation across the active layer. The fact that the experimental photocurrent does not saturate at the generation across the active layer, but gradually increases for higher effective voltage voltages, has been attributed to the field dependence of the generation rate.

## 4 Application of the Device Model Simulation to PSCs

### 4.1 PSCs

#### 4.1.1 PIN and NIP PSCs

As shown in Figure 1, metal halide perovskites have the generic chemical formula ABX_3_ with position A (green) the organic or inorganic cations occupy, B (gray) the metal cations and X (purple) positions halides, respectively.^91^ Organic–inorganic hybrid perovskite, such as MAPbI_3_ and MAPbBr_3_, was proven to be useful in photoenergy conversion by Miyasaka and co‐workers in 2009.^92^ Since then, PSCs have gained rapid progress, and an increasing amount of research is devoted to the device design. With many efforts, typical PSCs have been developed gradually with structures of organic–inorganic perovskite layers sandwiched between electron selective material, such as TiO_2_, mesoporous TiO_2_, PCBM, or C_60_, and a hole selective material, such as spiro‐OMeTAD, PEDOT:PSS [poly(3,4 ethylenedioxythiophene polystyrene) sulfonate]. The configuration of PSCs are various and are usually divided into the NIP and PIN structures. In the NIP configuration, the electron selective layer is deposited on the transparent conducting glass as the substrate, followed by the perovskite‐absorbing layer and (HSL) subsequently, whereas in the NIP configuration, the hole‐selective layer (ESL) is first deposited onto the substrate.^93^

PSCs exhibit a great number of distinctive features, owing to the semiconductor properties of perovskite materials. PSCs are exciton‐free cells. The excitons after light absorption have a binding energy of only ≈0.030 eV.^94^ Free carriers are generated immediately, Figure 5d, for most of the excitons dissociate very rapidly at room temperature. PSCs exhibit excellent charge and hole collection capabilities. Perovskites have been proven to be good electron and hole conductors with high carrier mobilities with ≈7.5 cm^2^ V^−1^ s^−1^ for electrons and ≈12.5 cm^2^ V^−1^ s^−1^ for holes.^95^ The charge collection capacity is further enhanced by the selective layers. The selective layers are designed to select charge carriers by allowing only electron or holes to be collected at one side. Charge loss is closely related to the device structure, micromorphology of the absorption layer and their preparation processes. Charge carrier loss happens in the bulk of the semiconductor layer, or at the interface between layers. There have been reports that indicated that the PEDOT:PSS/perovskite, SnO_2_ and TiO_2_ contacts constitute big surface recombination.^96^ Grain boundaries are believed to be a further source of defects that cause recombination in the perovskite films.^97^ Interface engineering and better control of the crystal formation greatly enhances the performance. The best achieved efficiency above 21.6% was obtained for PSCs.^98^

#### 4.1.2 Perovskite Tandem Solar Cells

The tandem solar cells structures have the potential to realize PCE beyond the Shockley–Queisser limit of single‐junction solar cells.^100^ For example, perovskite–perovskite tandems can be constructed by integrating the monolithic subcell of the perovskite layer with a bandgap *E*_g_ of 1.2 eV and subcells of the perovskite layer with a bandgap *E*_g_ of 1.8 eV (Figures 1e and 2e). The bandgap can be adjusted in this range from 1.2 to 2.3 eV.^99^ McMeekin et al. have reported that there is a perovskite material [HC(NH_2_)_2_]_0.83_Cs_0.17_Pb(I_0.6_Br_0.4_)_3_, with an optical bandgap of ≈1.74 eV.^101^ The rich perovskite material of various bandgap is the basis for designing various tandem solar cells.

The multilayer structure of the tandem device leads to greater complexity in designing the device. Rajagopal et al. demonstrated that the parameters of ESL will strongly influence the current matching of the subcells and *J*–*V* curves of the tandem devices. By engineering a precise tandem construction, they constructed a perovskite–perovskite tandem solar cell with a high *V*_OC_ of 1.98 V and a stabilized PCE of 18.5%.^102^ The PCE is still far from the theoretically achievable PCE of 36%. To further improve the PCE, losses of more detailed information on the multijunction solar cell should be assessed, and the potential of this all‐perovskite architecture needs to be illustrated. The device model simulation will help in designing precisely the parameter‐controlled tandem structures.

### 4.2 The Efficiency Limit of PSCs

Charge carrier and energy loss happen during any of the photovoltaic processes in PSCs. The photovoltaic processes can be divided into three successive steps: photoinduced charge generation, charge carrier separation, and efficient charge carrier extraction from the solar cells. Light absorption produces splitting of the Fermi level by exciting the electron to the conduction band and holes in the valence band. The splitting of the Fermi level provides the free energy that drives the cells. Charge carrier recombination is closely related to inefficient charge carrier separation and extraction, which deteriorates the free energy of the solar cells. Many factors, such as the nonohmic contact, trap states, direct band recombination, and surface recombination, cause free energy loss and *V*_OC_ reduction. The device model has been applied to describe the performance of the PSCs, which only consist of radiative recombination and surface recombination at the electrodes. The PCE limit set by many of the above processes is predicted.

**Figure 13:**
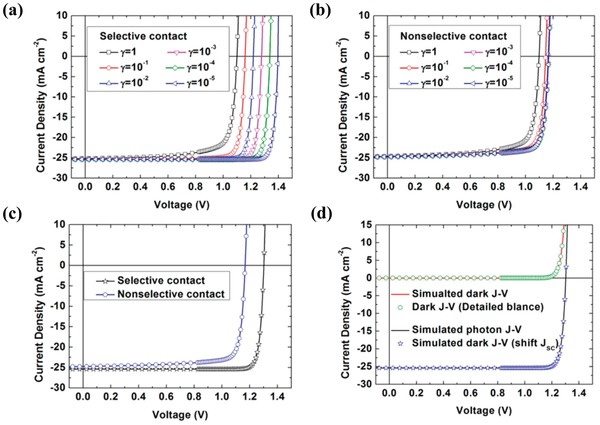
The modeled current *J*–*V* characteristics of PSCs with a) selective and nonselective contacts. b) The radiative recombination of PSCs with nonselective contact is modified by changing the reduction factor of γ. c) The radiative recombination of PSCs with selective contact is modified by changing the reduction factor of γ. d) *J*–*V* curves obtained by the drift‐diffusion model and detailed balance model, in the dark and under the illuminate respectively. Reproduced with permission.^103^ Copyright 2017, American Chemical Society.

Radiative recombination in bulk of the semiconductor and surface recombination at the contacts are two inevitable loss mechanisms. Surface recombination is caused by the collection of minority carriers together with the majority at the perovskite light‐absorbing layer and the FTO, ITO, and Au extracting contacts with a surface recombination velocity. Surface recombination set a PCE limit to the perovskite solar cells that is much lower than the limit set by the Shockley–Queisser theory. The device model simulation on the device with nonselective layers reveals that surface recombination gives an operation limit of the perovskite solar sell with *V*_OC_ of 1.17 V, *J*_SC_ of 24.74 mA cm^−2^ and PCE of 23.83, as shown in **Figure** **13**a. Fortunately, the surface carrier recombination can be inhibited by employing ESL and HSL in PSCs. The layers would inhibit the minority to the electrodes. The selective layer makes the surface recombination have an effect that can be neglected, and radiative recombination loss is the dominant role.

The bulk radiative recombination is one channel of energy loss. However, the device model results show that the PCE of 29.87% in the selective contact PSCs is achievable with the inhibition of trap‐assisted recombination. In the device model, bulk radiative recombination is expressed by Langevin's recombination coefficient β_L_ and a reduction factor γ, as in Equation (27). Under the nonselective contact, the varying γ from 1 to 10^−5^ has less impact on the performance of the PSCs, as shown in Figure 13b. This means that the operation PEC limit of PSCs with a nonselective contact can hardly be exceeded by reducing the radiative recombination. However, by reducing γ, significant performance enhancement of the selective contact PSCs has presented with *V*_OC_ of 1.30 V, *J*_SC_ of 25.38 mA cm^−2^ and PCE of 29.87% as shown in Figure 13c. As indicated above, the magnitude of the radiative recombination in perovskite materials is typically four‐orders of magnitude lower than that of Langevin's recombination.

PSCs with the selective layers and reduction factor γ show the prospect of PCE allowed by a detailed balance model, as shown in Figure 13d. The detailed balance model derives the efficiency limits that are allowed by the laws of thermodynamics. Reduction factor γ effects on the PCE explain why the perovskite semiconductor is an excellent choice for the absorption layer of solar cell devices. However, much more work should be done to further understand the effects of the traps, the electrodes, and the selective layers.

### 4.3 RSH Recombination in the Bulk and at the Surface

**Figure 14:**
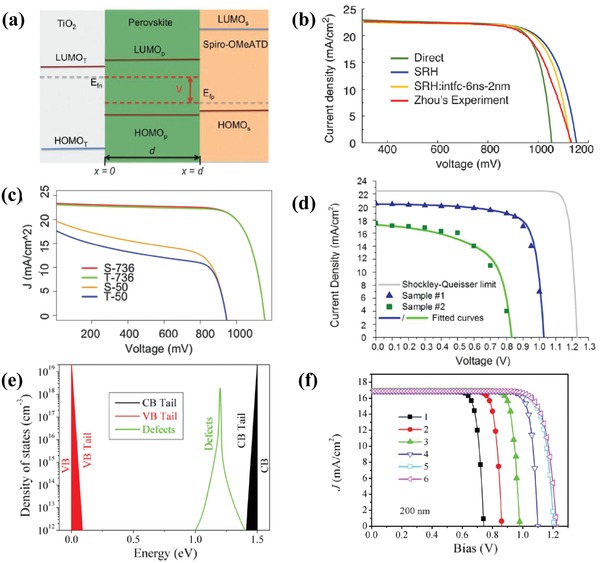
The simulation on the TiO_2_/MAPbI_3_/Spiro‐MeOTAD solar cell. a) Energy levels of TiO_2_/MAPbI_3_/Spiro‐MeOTAD solar cells. The output voltage of a solar cell is the potential difference between the electron quasi‐Fermi level at *x* = 0 and the hole quasi‐Fermi level at *x* = *d*. b) Comparison of the experiment results with the results of the direct recombination model and SRH models. c) Performances of solar cells with two types of structures and different lifetimes. S denotes the model with light coming from the Spiro‐MeOTAD layer; T denotes the model with light coming from the ITO layer. 736 denotes the charge lifetime of 736 ns and 50 denotes the charge lifetime of 50 ns. Reproduced with permission.^106^ Copyright 2016, Royal Society of Chemistry. d) *J*–*V* curves for the Shockley–Queisser limit, and simulation fit the device performances by two parameters of bulk defects density and interfacial defects density. Reproduced with permission.^104^ Copyright 2017, Elsevier B.V. The grain Boundary defects effects are studied. e) Schematic band diagram for the perovskite absorber layer used in this investigation. f) Current density–voltage characteristics of PSCs due to variation in grain boundary defect density, Here the traces 6–1 correspond to grain boundary defect density as 3 × 10^12^, 3 × 10^14^, 3 × 10^16^, 3 × 10^18^, 3 × 10^20^, 3 × 10^22^ cm^−3^ respectively. Reproduced with permission.^108^ Copyright 2018, Elsevier B.V.

With the surface recombination inhibited by selective layers, the trap/defect dependent recombination in the bulk of the semiconductor or at the surface emerge as the primary loss mechanism in many PSCs.^104^, ^105^, ^106^, ^107^, ^108^ Thus, traps/defects are the main obstacles to achieve the maximum PCE of the PSCs limit set by the laws of thermodynamics. The traps are distributed in the bulk of the semiconductor and at the surface between the selective layer and the perovskite layer. The device model methods have been applied to model the charge carrier loss mechanisms in PSCs with traps, and the results have been verified by experimental data. The Shockley–Read–Hall (SRH) theory is adopted to describe the traps/defects recombination in the device model of PSCs. A bulk defect density blow 1 × 10^15^ cm^−3^ and interface defect density less than 1 × 10^9^ cm^−2^ for the sample will guarantee a PCE higher than 15.7%. For example, Zhou et al. implemented a simulation on the TiO_2_/MAPbI_3_/Spiro‐MeOTAD solar cells with direct recombination and RSH recombination, as shown in **Figure** **14**a.^106^ By comparing the fill factors with the experiment in Figure 14b, it was found that the RSH recombination model gives an FF of 76.15%, which is closer to FF (75.07%) in the experiment, while the direct band‐to‐band recombination model gives a fill factor as high as 79.89%. In their modeling, the intrinsic charge recombination rate is proportional to densities of electrons and holes by the Langevin theory; traps and defect recombination are described by the SRH theory. They withdrew the charge carrier lifetime due to different recombination mechanisms from the experiment data. In their interpretation, intrinsic bulk recombination induced the carrier lifetime by ≈736 ns, and the interface recombination induced a carrier lifetime of ≈0.1–10 ns.

Compared with the defect dependent recombination in the bulk of the semiconductor, interface recombination has a greater influence on the *V*_OC_, the fill factor and the PCE. First, almost all perovskite layers have better conductivity than organic or oxide ESL/HSL, resulting in an abundant charge carrier density at the interface between ESL/HSL and perovskite layers. Second, an unbonded electron at the interface causes a higher density of defects and traps. Moreover, the discontinuity of the conduction band and valence band hinder the charge carrier collection and enhances the charge recombination at the surface. Figure 14c shows the performance of solar cells with two types of structures and different lifetimes. S denotes the model with light coming from the Spiro‐MeOTAD layer, T denotes the model with light coming from the ITO layer, 736 means the charge lifetime of 736 ns, and 50 means the charge lifetime of 50 ns. They revealed that a shorter charge carrier's lifetime (50 ns) by interface recombination has more influence on the *V*_OC_, fill factor and PCE, and the cells that are not well fabricated are attributed to enhanced interface recombination. Olyaeefar et al. have also proven the importance of trap recombination by fitting the device performance well with two parameters of the bulk defect density and the interfacial defect density at TiO_2_/MAPbX_3_,^104^ as in Figure 14d. A bulk defect density of 7 × 10^15^ cm^−3^ and an interface defect density of 1 × 10^9^ cm^−2^ are used to fit the *J*–*V* curves of the device sample with a higher PCE of 15.7%.^109^ For the sample with lower PCE of 8.6%, the bulk and interface defect densities are set as 5 × 10^16^ cm^−3^ and 3 × 10^11^ cm^−2^.^21^

The trap density is reported to relate to the perovskite crystallinity. The device model simulation verified that the reported trap density of the high‐quality crystallized film is below 10^15^ cm^−3^. Iftiquar and Yi introduced the grain boundary defects to the device model and the effects of the trap due to the grain boundary were simulated by AFORS‐HET simulation programs.^108^ In Figure 14e, the grain boundary defects is introduced as conduction band tails near valence, rather than midgap defects, and the grain boundary equivalent of the volume defect density varied from 3 × 10^12^ to 3 × 10^22^ cm^−3^, while all other material and device parameters were kept unchanged. The simulation shows that the *J*_SC_ remained consistent with the change in the grain boundary defect density, while *V*_OC_ decreases steadily. The results also show a systematic reduction efficiency due to the increase in the grain boundary defect density, while the short‐circuit current is unchanged with the varied trap density, as shown in Figure 14f. The simulation proves the major role of RSH recombination at the selective layer interface.

### 4.4 HSL and ESL Effect

HSL and ESL are widely used in PSCs to avoid exposure of the perovskite layer to the FTO, ITO, metal extracting contacts. Parameters should be chosen to examine the effects of selective layers on the performance of the PSCs. Conduction band offset (CBO), charge carrier mobility, and acceptor density are typical parameters, which have a significant effect on the device performance. The device model analysis has been performed, and rules have been revealed to design an optimum HSL and ESL for efficiency and device stability.

**Figure 15:**
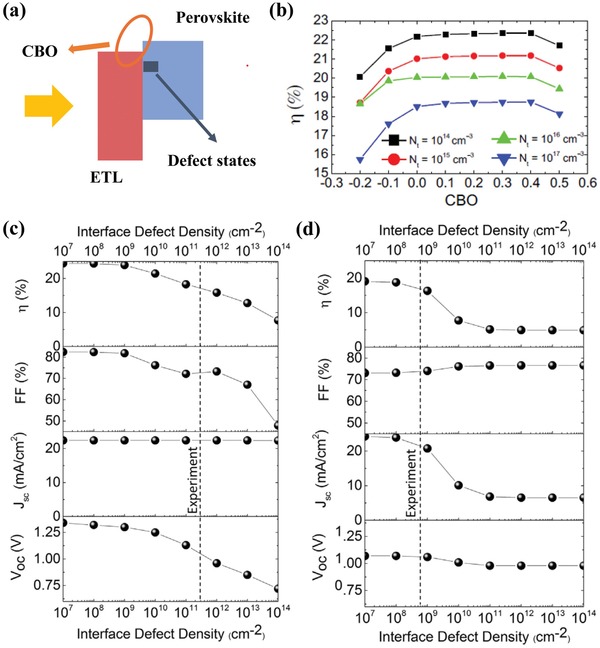
a) The surface electronic structure between ESL and perovskite layer with CBO = χ_perovskite_ − χ_ESL_, χ is the electron affinity. Variation of device characteristics as a function of defect density at b) CBO; reproduced with permission,[qv: 107a] Copyright 2016, IEEE; c) HSL/perovskite interface; and d) ESL/perovskite interface. Reproduced with permission.[qv: 107b] Copyright 2018, Elsevier B.V.

HSL and ESL introduced defects at the interface between the selective layer and perovskite. The device model simulation found the correlation of the selective layer defect recombination to the surface electronic structure, which can help in the design of the selective layers. The simulation suggested ESL with an electron affinity of 0.1–0.3 eV is smaller than the electron affinity of perovskite layers in the NIP PSCs, and HSL with the valance band of 0.1–0.3 eV being bigger than the valance band of perovskite layers in the PIN PSCs.^107^ As shown in **Figure** **15**a, the conduction band offset CBO = χ_perovskite_ − χ_ESL_, χ is the electron affinity. If CBO is negative, then the electronic structure assists the electron capture by defect states, which cause additional power loss in the NIP PSCs. The picture is verified by the device model simulation on the device with SRH defect recombination. Figure 15b shows the effect on PCE of CBO at the ESL/perovskite interface, which is obtained by Aryal et al. with device model simulations. The ESL electron affinity varied from 4.1 to 3.4 eV, thus corresponding a varied CBO. It was found that the highest simulated device efficiency occurs for the CBO of 0.1 to 0.4 eV with the absorber layer. The *J*–*V* curves of the negative CBO depend on the amount of interfacial recombination and show a reduction in the voltage, and fill factor. Based on the simulation, high electron affinity materials have been assessed as alternatives to replace TiO_2_ as the electron transport layer. They argued that bandgap tunable ZnOS with varying oxygen content is the best choice, thus resulting in an optimal CBO with the perovskite absorber and the best device performance.

The device model simulation also revealed typical features of the dependence of *V*_OC_, fill factor, and PCE on the defect density. Chouhan et al. adopted the Solar Cell Capacitance Simulator(SCAPS) to simulate the variation of device parameters as a function of defect density in typical NIP devices fabricated with the FTO/c‐TiO_2_/m‐TiO_2_/Perovskite/Spiro‐MeOTAD/Au architecture.^107^ They revealed that the defect density at the HSL/perovskite interface has a substantial impact on the *V*_OC_, as shown in Figure 15c, while the defect density in the perovskite and ESL/perovskite interface has a significant effect on short‐circuit current density, as shown in Figure 15d. In their models, the CBO at the HSL/perovskite is 0.29 eV, defect recombination is not facilitated, and the defect state at HSL/perovskite has a week impact on the short‐circuit current density. However, the CBO at the ESL/perovskite is −0.1 eV; by analysis, the trap state recombination is enhanced, thus resulting in a major impact on the short‐circuit current density. The simulation is verified by fitting the 17.5% PCE efficient PSCs with a bulk defect density of 10^13^ cm^3^.

**Figure 16:**
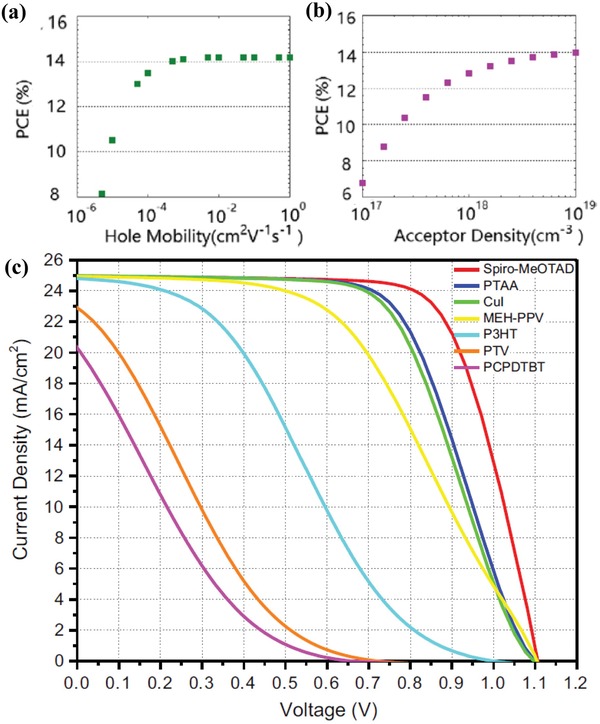
Effects of HTM layer characteristics, a) hole mobility, b) acceptor density, on power conversion efficiency (PCE) of perovskite solar cells, and c) *J*–*V* curve of with various HSL candidates in device simulation. Reproduced with permission.^110^ Copyright 2016, Elsevier Ltd.

In modeling the physical processes, deep insights have been obtained, which helps in determining the characteristic of the layers. Tan et al. adopted SCAPS solar cell simulator to design the TiO_2_/perovskite layer/HSL layer solar cells.^110^ They examine the effects of HSL PCE by two typical parameters, hole mobility, and acceptor density. **Figure** **16**a gives the simulation results for PCE as a function of the hole mobility. It was observed that the PCE showing a maximum saturation point (14.19%) at the hole mobility is 1 × 10^−2^ cm^2^ V^−1^ s^−1^. Figure 16b shows an elevated PCE of the simulated solar cells with an increase of the acceptor density. The results explain the p‐type dopant effects in the HSLs on solar cell performance, which is commonly used in the device fabrication. The result is consistent with the belief that doping in the ESL and HSL increases the hole mobility and charge density, thus resulting in improved device performance. They also examined several commonly used HSLs, such as Spiro‐MeOTAD, poly(thiophene‐3‐acetic acid)(PTAA), CuI, poly [2‐methoxy‐5‐(20‐ethyl‐hexyloxy)‐1,4‐phenylenevinylene](MEH‐PPV), P3HT, poly(2,5‐thienylene vinylene)(PTV) and poly[2,6‐(4,4‐bis‐(2‐ethylhexyl)‐4H‐cyclopenta[2,1‐b;3,4‐b0]‐dithiophene)‐alt‐4,7‐(2,1,3‐benzothiadiazole)](PCPDTBT). Figure 16c presents photocurrent density–voltage (*J*–*V*) curves for cells with various HSL candidates in the device simulation. The simulator indicated that the cell with typical Spiro‐MeOTAD as an HSL layer presents the highest 20% PCE. It is worth noting that PTAA and CuI exhibit better property (17.4%) than other HSLs, which give promise as a potential HSL. The simulation also focuses on the device performance parameters as a function of absorber thicknesses. It was shown that *J*_SC_ increases apparently with the increasing absorber thickness and reaches the maximum value of 24 mA cm^−2^ at ≈900 nm thickness. *V*_OC_ increases to an optimal value (1.055 V) at 500–600 nm thickness and then decreases sharply. An optimal absorber thickness (600–700 nm) is derived from the power conversion efficiency.

### 4.5 *J*–*V* hysteresis in PSCs

**Figure 17:**
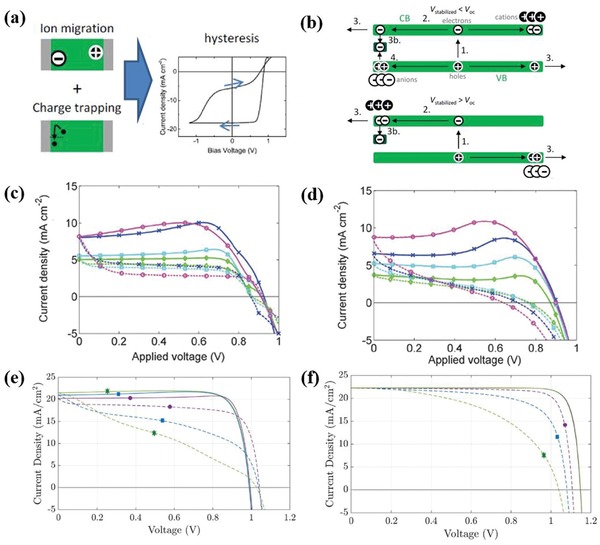
a) Schematic diagram of causes of the hysteresis effects: the combination of mobile ions and electron traps at the ESL/perovskite interface. b) Schematics of the charge flow in the conduction band (CB) and valence band (CV) of the perovskite in case *V*_stabilized_ < *V*_OC_ and *V*_stabilized_ > *V*_OC_ with 1. charge generation; 2. charge transport; 3a. charge extraction; 3b. charge trapping; 4. trap‐assisted charge recombination. Reproduced with permission.^119^ Copyright 2015, American Chemical Society. c) Calculated *J*–*V* curves. d) Measured *J*–*V* curves. Solid lines show the 1.2 to 0 V scan; broken lines show the 0 to 1.2 V scan. Scan rates are 1 V s^−1^ (magenta, circles), 500 mV s^−1^ (blue, crosses), 250 mV s^−1^ (cyan, filled squares), and 100 mV s^−1^ (green, diamonds). Reproduced with permission.^122^ Copyright 2018, Cambridge University Press. *J*–*V* curves scan rates of 20 mV s^−1^ (purple, circles), 60 mV s^−1^ (blue, squares) and 100 mV s^−1^ (green, stars); Solid lines represent the initial reverse scans e) losses that are dominated by bulk recombination f) losses that dominated by interfacial recombination. Reproduced with permission.^4^ Copyright 2019, Royal Society of Chemistry.

The hysteresis phenomena observed in many perovskite cells stimulated extensive theoretical and experimental research due to their remarkable features. Hysteresis phenomena are the current density–voltage hysteric response between forward and reverse scans to characterize the cells. It exhibits a memory effect on operations to the devices. Typically, a higher PCE is displayed in the reverse scan from an open‐circuit condition to a short‐circuit condition, as shown in **Figure** **17**a, and a reduction of the PCE of 10% or more is observed during the forward scan from the short‐circuit condition to an open‐circuit condition in solar cells in many configurations.^111^, ^112^ The *J*–*V* hysteresis behavior of the PSCs is a nonlinear phenomenon, which is influenced by the scan direction, scan rate, and voltage range during the photocurrent characterization.^113^ The preparation and the architecture of PSCs are also reported to present a great influence on the *J*–*V* hysteresis behaviors.^114^ The varying temperature and light intensity were also found have an influence on the hysteresis behaviors.^115^ Fullerene passivation of the perovskite/ETL interface^116^ and replacing TiO_2_ with a SnO_2_ ETL^117^ were reported to eliminate the hysteresis phenomena. Kim et al.^118^ and Richardson and co‐workers^4^ gave a very detailed review of the experimental findings.

To reproduce the density‐voltage hysteric response, many of its features theoretically have an irreplaceable role in defining the cause. Although some theories have been put forward to explain the strange behaviors, the device model simulation is the method that reproduces many of the hysteresis features by modeling the coupling of slow ion dynamics and the charge carrier surface recombination at the ESL/perovskite. Reenen et al.^119^ and Richardson et al.^120^ provided initial works that use device models that incorporate ion migration to explain hysteresis in the *J*–*V* curves of the PSCs. They point out that severe spatial and temporal stiffness arise, which is a challenging numerical solution for the appropriate partial differential equations. Upon realizing that the stiffness is introduced by narrow ionic Debye layers and that there is large disparity between the timescales of ion migration and a much faster charge carrier motion, Richardson et al. used the method of matched asymptotic analysis to analyze the problem.^120^ Courtier et al. also present a numerical method that is capable of accurately solving the extremely stiffness device model equations.^121^

Snaith et al. have included ion migrations into the device models.^111^, ^119^ As a free electron/hole charge carrier, the ion movement is modeled by the drift‐diffusion equation, but with an ion mobility that is six orders lower than the mobility of the free electron. Their work first links ion movements to the recombination of charges, thereby giving a clear interpretation of the phenomenon. As shown in the upper panel of Figure 17b, if the device working at voltage *V*_stabilized_ < *V*_OC_ lasts for a long enough time interval, then anions drift and distribute at the ESL/perovskite interface and are driven by the internal electric field. The accumulation of anions can localize part of the hole charge carriers. The localized holes recombine with electrons through trap/defect states at the interface, thus resulting in a reduction of the photocurrent. However, if the device working at voltage *V*_stabilized_ > *V*_OC_ lasts for a long enough time interval, anions drift and distribute at the HSL/perovskite interface, and holes are collected with a bigger efficient, without less chance to recombine with electrons through trap/defect states at the ESL/perovskite interface. The theory gives a reasonable explanation for the hysteresis phenomena. Under the reverse scan from the open‐circuit voltage condition to a short‐circuit condition, anions are prepared at the HSL/perovskite interface, anion migration lags the scan process, and fewer anions accumulate at the ESL/perovskite interface, which causes no further recombination of the charge carriers. However, under the forward scan from the short‐circuit condition to an open‐circuit condition, anions are prepared at the ESL/perovskite interface, there is a delay of anion migration to voltage scanning, which results in a reduction of the photocurrent by recombination.

The device model of slow ion migration reproduces the typical hysteresis characteristics in perovskite solar cells.^120^, ^122^ The simulation reproduces the features of the influence by the scan rate on *J*–*V* hysteresis curves (Figure 17c), which have the corresponding measured *J*–*V* hysteresis curves (Figure 17d). As shown by both the simulated and measured *J*–*V* curves, for slower scan rates, the vacancies have more time to respond to the change in applied voltage than to faster scans. The photocurrent dependence less on the history of the electronic bias, and small hysteresis is present, as shown by green diamonds lines under Scan rate 100 mV s^−1^. At the other extreme, if the scan is sufficiently fast, then the ions do not have a chance to respond, so there is a vacancy distribution lag behind, which results in a recombination dependence on the history of the electronic bias history; bigger hysteresis is present as the magenta, circle lines at the scan rates are 1 V s^−1^.

Recently, Richardson and co‐workers presented a high‐performance numerical method to solve the model for coupled ion vacancy motion and charge transport in a three‐layer planar perovskite solar cell.^4^ The accurate solution can describe how the potential drops are apportioned between the ESL, perovskite and HSL. The ion distributions induced internal potential distribution affects charge carrier recombination and, consequently, the current. Their methods can investigate how properties of the selective layers influence the extent of *J*–*V* hysteresis. The results demonstrate that the replacement of the standard transport layer materials (spiro‐OMeTAD and TiO_2_) by materials with lower permittivity and/or doping leads to a shift in the scan rates at which hysteresis is most pronounced to rates that are higher than those that are commonly used in the experiment. These results provide a cogent explanation for why organic electron transport layers can yield seemingly hysteresis‐free devices but, nevertheless, exhibit hysteresis at low temperatures. What is more interesting is that their simulations can be used to classify features of the *J*–*V* curves that distinguish between cells in which the charge carrier recombination occurs predominantly at the transport layer interfaces and those where it occurs predominantly within the perovskite. Such studies have the potential to guide future cell development and to assess cell degradation as a tool. Figure 17e,f shows that *J*–*V* curves for two representative cells have energy losses that are dominated by bulk recombination and interfacial recombination, respectively. Three distinct features are present to distinguish bulk recombination and interfacial recombination. The first feature is the current maximum on the reverse scans of the two slower *J*–*V* curves in Figure 17e. Another feature that can be attributed to bulk recombination is that of a noticeable drop‐off in the current just after the switch in the scan direction at a short circuit. The third feature is the significantly depressed *V*_OC_ in Figure 17f of the device with dominated interfacial recombination.

### 4.6 Tandem Solar Cells

Tandem solar cells have been used as an efficient approach to overcome the Shockley–Queisser limit and achieve high efficiency. However, high‐efficiency tandem solar cells require many device optimization studies and ask for heavy experimental works. Device modeling for tandem solar cells turned out to be a practical approach to accelerate the optimization procedures.

One of the challenges of modeling tandem solar cells is the connection of subcells. Better treatment of the carrier transport at the interfaces is indispensable. Thermionic emission, intraband tunneling, and the trap‐assisted tunneling mechanism must be considered to describe the junction behavior correctly. At present, several solar cell modeling codes, such as the free programs PC1D, SCAPS, AFORS‐HET, and AMPS, have been adopted by incorporating the tunneling mechanism. These programs have great potential application with respect for designing the thin film tandem cell.^124^, ^125^, ^126^

**Figure 18:**
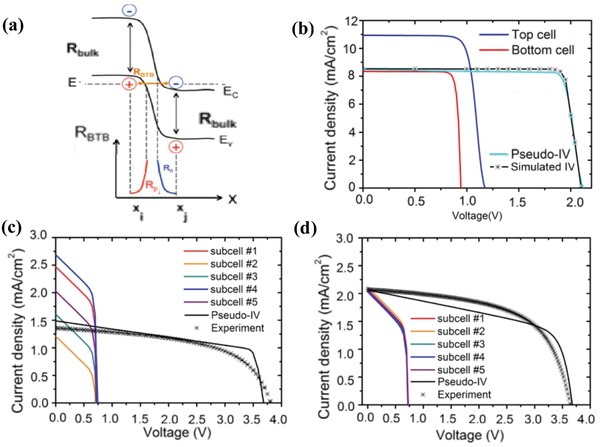
a) Schematic diagram on the nonlocal band to band tunneling models. *E*_c_, *E*_v_ represent conduction band and valence band, respectively. *R*_bulk_ denotes the bulk recombination caused by nonradiative defect related SRH recombination or radiative recombination. b) Simulated subcell IV curves and corresponding pseudo‐IV curve for the InGaP/GaAs dual‐junction cell. The subcell analysis only consumes seconds of computation time with a common computer, and the generated pseudo‐IV curve is very close to the curve simulated without simplifying the tunneling junction. c) Simulated and experimental IV curves for a prototype organic tandem cell before optimization. Each subcell is fabricated of MoO_3_/DBP(10 nm)/C70(10 nm)/BCP(7 nm) and interconnected via a 0.5 nm Ag recombination layer. Strong optical interference effects occur within these thin layers and lead to severe current mismatches between subcells. d) Simulated and experimental IV curves for the organic tandem cell with the optimized thicknesses. The optimization is achieved by increasing the thicknesses of subcells #2 and #3 and decreasing the thicknesses of subcells #1 and #4. Reproduced with permission.^123^ Copyright 2018, IEEE.

Recently, Liu et al. reported the enhancements to wxAMPS. The program has already included intraband tunneling and trap‐assisted tunneling. They further incorporated the nonlocal band‐to‐band tunneling model to describe the device behavior of tandem solar cells better.^123^ The code is now suitable to model tandem solar cells of different types. **Figure** **18**a shows that the nonlocal band‐to‐band tunneling models with the tunneling probability depends on the whole potential profile across the tunneling region. The nonlocal model reflects the realistic nonlocal phenomena of band‐to‐band tunneling, and the adoption of the tunneling model is thought to render the wxAMPS as more suitable for simulating and optimizing inorganic/organic hybrid tandem solar cells. Yiming et al. also developed a subcell analysis feature to facilitate batch simulations for tandem cells.

To verify the models and subcell analysis feature, they applied the methods to analyze the InGaP/GaAs dual‐junction cell. Figure 18b shows the simulated subcell IV curves and the corresponding pseudo‐IV curve for the InGaP/GaAs dual‐junction cell. When implementing a subcell analysis, the subcell is simulated separately. The pseudo‐IV curve for the whole cell is then obtained by the subcell IV curves. The subcell analysis only consumes seconds of computation time with a common computer, and the generated pseudo‐IV curve is very close to the curve simulated without simplifying the tunneling junction. The *V*_OC_ from pseudo‐IV is slightly higher (<5 mV) than that of simulated IV, which is the effect of tunneling. This subcell analysis feature avoids the complexity of the tunneling model. The simulation converges fast, and the outputs will be accurate, as long as the tunneling junction is not the bottleneck of the photocurrent. The simulator is further used to optimize the thicknesses of a prototype organic tandem cell with subcells fabricated with MoO_3_/DBP(10 nm)/C_70_(10 nm)/BCP(7 nm) and interconnected via a 0.5 nm Ag recombination layer. As shown in Figure 18c,d, the photocurrent is promoted from 1.5 to 2.5 mA cm^−2^ by increasing the thicknesses of subcells #2 and #3 and decreasing the thicknesses of subcells #1 and #4.

Greater efforts have also been implemented to optimize the tandem solar cells by using the device model. Ramli et al. have used the solar cell capacitance simulator structures (SCAPS‐1D) to model the cell configuration of the Si‐perovskite tandem solar cells.^126^ The *V*_OC_, fill factor and efficiency influenced by the thickness variation of the MAPbI_3_ are simulated, which indicated that the highest values of 0.8178 V and 27.27% occur when the MAPbI_3_ thickness is 300 nm. The Si‐perovskite tandem solar cells were also investigated by varying the donor dopant concentrations of MAPbI_3_ and TiO_2_ from 10^12^ to 10^18^ cm^−3^ to optimize toward better solar cell performance. A saturated point with a value of 27.19% for the donor dopant concentration range of 10^16^–10^18^ cm^−3^ was reached with an increasing dopant concentration of MAPbI_3_ from 10^12^ to 10^16^ cm^−3^.

## 5 Conclusion

In this review, we discuss the importance of the device model simulation for thin film solar cells ranging from organic to perovskite. The results show that the device model simulation establishes a direct relationship between the observable current–voltage curves and related microphysical processes, and has a deep understanding of the working mechanism of thin film solar cells. By comparing the application of the device model method in the OSCs and PSCs simulation, the performance of the device is demonstrated from the point of view of the microphysical process. We have concluded that the electronic processes at the contacts and interfaces of all layers play an important role in all devices. The results show that the *J*–*V* curve is sensitive to the metal/organic interface in OSCs, and the charge aggregation in PSC is sensitive to the selective layer/perovskite interface. The recombination and dissociation of excitons in organic materials at the donor–acceptor interface should be modeled to generate free charge carriers. The space charge effect is a general factor hindering the charge carrier collection in OSC. The space charge screen generates a space‐charge‐limited current with secondary voltage function in the electric field of semiconductor materials. Space charges can accumulate under ohmic contact conditions, or there is an imbalance between the electron and hole charge carrier mobility in OSC. In contrast, exciton‐free perovskite materials, the space charge‐free effect, and reduced bimolecular recombination ensure the high PCE of PSCs. The effects of traps and tunneling on the current flow have been widely simulated in OSCs, but their effects on PSC performance have rarely been revealed. Transient models of photovoltaic and photocurrent have also been well established in OSC modeling and can be extended to PSCs.

These topics are still traditional and widely studied subjects that lay a foundation for the establishment of device model methods. In the case of 23.3% PCE, we should further understand the fine electronic processes of PSCs, such as the electronic processes at grain boundaries, the electronic processes in materials with traps and additives, the electronic processes across thin interlayers, and the electronic processes of multifilm structures. In addition, the combination of these factors affects the performance of the device, thus resulting in a large variable space in the actual device design. These topics are now common in new PSCs, and understanding this mechanism is only in its infancy. The trap density of high‐quality crystalline film is less than 10^15^ cm^−3^, which is the main obstacle for realizing the maximum PCE of PSC and is restricted by thermodynamic law. To explain *J*–*V* hysteresis, a coupled model of ESL/perovskite surface recombination and slow ion motion was established. At the same time, the relationship between the defect recombination in the selective layer and the electronic structure of the interface surface was revealed, and more work needs to be done. The detailed balance theory and the equivalent circuit theory cannot reveal the subtle process; they can only give an ideal description of the battery performance. To solve this problem, the device model method needs to be improved to simulate the transient characteristics of solar cells, such as transient photocurrent and photovoltage, capacitance voltage and the impedance spectrum. Combining the device model simulation and the electrical characteristics of thin film solar cells, the fine electronic process of PSCs and its influence on performance will be revealed.

In conclusion of this review, the device model is a powerful tool for predicating the characteristics of the thin device from organic to perovskite. By linking the observable current–voltage curves directly to each of the relevant microscopic physical processes, the method exhibits a concept of computer‐assisted design of the thin film solar cells. The device model simulation will be helpful in the mass production of the versatile structural thin film devices.

## Conflict of Interest

The authors declare no conflict of interest.
